# Aberrant hypermethylation-mediated downregulation of antisense lncRNA ZNF667-AS1 and its sense gene ZNF667 correlate with progression and prognosis of esophageal squamous cell carcinoma

**DOI:** 10.1038/s41419-019-2171-3

**Published:** 2019-12-05

**Authors:** Zhiming Dong, Shengmian Li, Xuan Wu, Yunfeng Niu, Xiaoliang Liang, Liu Yang, Yanli Guo, Supeng Shen, Jia Liang, Wei Guo

**Affiliations:** 1 Laboratory of Pathology, Hebei Cancer Institute, the Fourth Hospital of Hebei Medical University, Shijiazhuang, Hebei China; 2 Department of Digestive Internal, the Fourth Hospital of Hebei Medical University, Shijiazhuang, Hebei China

**Keywords:** Oesophageal cancer, Tumour biomarkers

## Abstract

Natural antisense lncRNAs can interfere with their corresponding sense transcript to elicit concordant or discordant regulation. LncRNA ZNF667-AS1 and its sense gene ZNF667 were found to be downregulated in esophageal squamous cell carcinoma (ESCC) tissues by RNA sequencing; however, the exact roles of both genes in ESCC occurrence and development have not been clarified. This study was to investigate the expression patterns, epigenetic inactivation mechanisms, function, and prognostic significance of ZNF667-AS1 and ZNF667 in ESCC tumorigenesis. Frequent downregulation of ZNF667-AS1 and ZNF667 was detected in esophageal cancer cells and ESCC tissues. The expression levels of ZNF667-AS1 and ZNF667 were significantly reversed by treatment with 5-Aza-dC and TSA in esophageal cancer cell lines. The CpG sites hypermethylation within proximal promoter influenced the binding ability of transcription factor E2F1 to the binding sites and then affected the transcription and expression of ZNF667-AS1 and ZNF667. Overexpression of ZNF667-AS1 and ZNF667 suppressed the viability, migration, and invasion of esophageal cancer cells in vitro. Overexpression of ZNF667-AS1 increased mRNA and protein expression level of ZNF667. ZNF667-AS1 interacts with and recruits TET1 to its target gene ZNF667 and E-cadherin to hydrolyze 5′-mc to 5′-hmc and further activates their expression, meanwhile, ZNF667-AS1 also interacts with UTX to decrease histone H3K27 tri-methylation to activate ZNF667 and E-cadherin expression. Furthermore, ZNF667-AS1 or ZNF667 expression and promoter methylation status were correlated with ESCC patients’ survival. Thus, these findings suggest that ZNF667-AS1 and ZNF667 may act as tumor suppressors and may serve as potential targets for antitumor therapy.

## Introduction

Esophageal cancer is the ninth most common cancer in the world, and the sixth most common cause of cancer-related death^1,2^. With high mortality and poor prognosis, esophageal cancer includes two main histological types: esophageal adenocarcinoma (EAC) and esophageal squamous cell carcinoma (ESCC). Globally, ESCC is the most common histological subtype of esophageal cancer, particularly in high-incidence areas of eastern Asia and in eastern and southern Africa^3^. The incidence of ESCC in China is higher, especially in Shanxi, Henan, Hebei, and other areas of the Taihang Mountains in northern China^4^. Family aggregation occurs in these high-risk areas; however, the exact etiology and mechanisms of ESCC remain unclear.

Long noncoding RNAs (lncRNAs), which often lack protein-coding capabilities, have recently been investigated as oncogenes or tumor suppressors by regulating many biological processes in human cancers via diverse mechanisms^5,6^. A particular group of lncRNAs are natural antisense transcripts (NATs). NATs are transcribed in the opposite direction to protein-coding transcripts, and are widespread in eukaryotes^7^. NATs overlap with sense genes, their promoters, or other regulatory regions. Despite being processed mostly from protein-coding loci, they frequently display distinct characteristics compared with their sense partners^8^. NATs may be expressed at lower levels than sense transcripts and often demonstrate high tissue specificity. NATs can predominantly interfere with their corresponding sense transcript to elicit concordant and discordant regulation in cis, or can regulate transcripts from other genomic loci to act in trans^9^. However, the exact molecular mechanisms triggered by NATs in the context of these regulatory roles are currently poorly understood.

The roles of NATs in the occurrence and development of ESCC need to be clarified. By retrievaling literature and databases, and further bioinformatics analyses, lncRNA ZNF667-AS1 and its sense gene ZNF667 were found to be downregulated in ESCC tissues by RNA sequencing^10^. The expression of ZNF667-AS1 was reported to be closely associated with the mortal, finite lifespan phenotype^11^. ZNF667-AS1 was expressed in all normal finite lifespan human cells, and was lost in immortalized human mammary epithelial cells (HMEC) and in 22 of 33 of The Cancer Genome Atlas (TCGA) cancer types, including cervical cancer, cancers of esophagus, stomach cancer, bile duct carcinoma, mesothelioma, sarcoma, and uterine carcinosarcoma^11–14^. ZNF667-AS1 expression is gradually downregulated after spinal cord injury (SCI), and ZNF667-AS1 inhibits the inflammatory response and promotes SCI recovery via suppressing the JAK-STAT pathway^15^. Moreover, ZNF667-AS1 gene silencing in various tumor cells was detected to be attributed to DNA hypermethylation of its promoter CpG island^11,12,14,16^. ZNF667-AS1 was also identified to be a hypermethylated lncRNA in most of the tumor types in a study characterizing the epigenetic landscape of genes encoding lncRNAs across 6475 tumors and 455 cancer cell lines^17^. As a head-to-head sense gene with ZNF667-AS1, ZNF667 is a coding gene and the transcription start site is 473 bp away from ZNF667-AS1. Sharing with the same CpG islands, whether expression of ZNF667-AS1 and ZNF667 are both regulated by DNA methylation in ESCC is unclear, furthermore, and the roles of both genes and the regulatory effect of each other in ESCC need to be clarified.

## Results

### Decreased expression of ZNF667-AS1 and ZNF667 in esophageal cancer cells and ESCC tissues

**Fig. 1:**
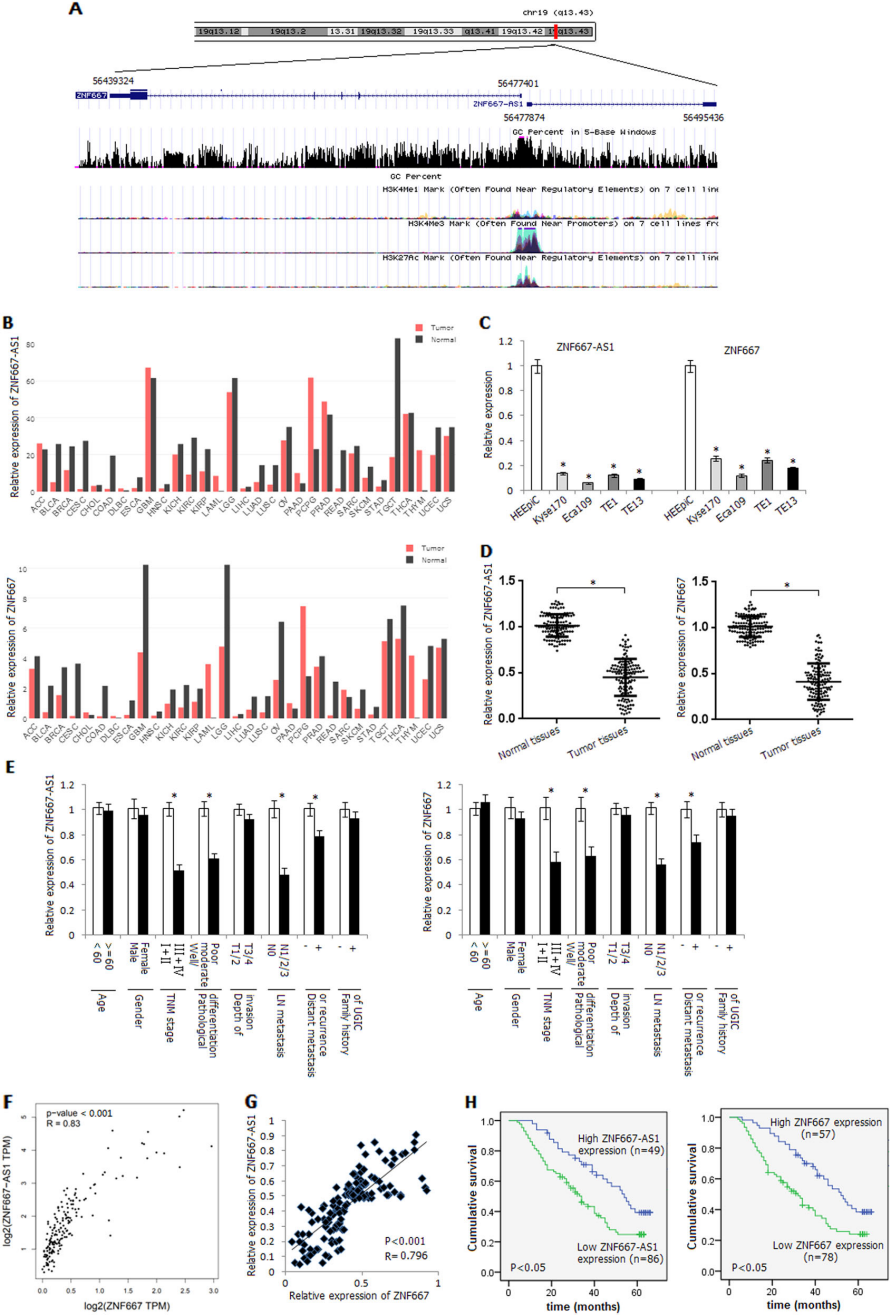
Expression status and survival analysis of ZNF667-AS1 and ZNF667 in ESCC. **a** Schematic representation of the genomic organization of ZNF667-AS1 and ZNF667. **b** Relative expression of ZNF667-AS1 and ZNF667 in various tumor types in GEPIA data set. ACC adrenocortical carcinoma, BLCA bladder urothelial carcinoma, BRCA breast invasive carcinoma, CESC Cervical squamous cell carcinoma and endocervical adenocarcinoma, CHOL cholangio carcinoma, COAD colon adenocarcinoma, DLBC lymphoid neoplasm diffuse large B-cell lymphoma, ESCA esophageal carcinoma, GBM glioblastoma multiforme, HNSC head and neck squamous cell carcinoma, KICH kidney chromophobe, KIRC kidney renal clear cell carcinoma, KIRP kidney renal papillary cell carcinoma, LAML acute myeloid leukemia, LGG brain lower grade glioma, LIHC liver hepatocellular carcinoma, LUAD lung adenocarcinoma, LUSC lung squamous cell carcinoma, OV ovarian serous cystadenocarcinoma, PAAD pancreatic adenocarcinoma, PCPG pheochromocytoma and paraganglioma, PRAD prostate adenocarcinoma, READ rectum adenocarcinoma, SARC sarcoma, SKCM skin cutaneous melanoma, STAD stomach adenocarcinoma, TGCT testicular germ cell tumors, THCA thyroid carcinoma, THYM thymoma, UCEC uterine corpus endometrial carcinoma, UCS uterine carcinosarcoma. **c** Relative expression level of ZNF667-AS1 and ZNF667 in human normal esophageal epithelial cells HEEpiC and four esophageal cancer cell lines detected by qRT-PCR method. **d** Relative expression level of ZNF667-AS1 and ZNF667 in ESCC tissues and corresponding normal tissues. **e** Relative expression level of ZNF667-AS1 and ZNF667 in different subgroups. **f** The correlation between the expression level of ZNF667-AS1 and ZNF667 in ESCA in GEPIA data set. **g** The correlation between the expression level of ZNF667-AS1 and ZNF667 in ESCC tissues. **h** The influence of expression level of ZNF667-AS1 or ZNF667 on ESCC patients’ survival. *P < 0.05.

ZNF667-AS1 and ZNF667 genes are head-to-head distributed genes, and there are 473 bases interval between the transcriptional start sites of them (Fig. 1a). ZNF667-AS1 has no coding potential (Supplementary Fig. S1). We first scanned the relative expression level of ZNF667-AS1 and ZNF667 in various tumor types in Gene Expression Profiling Interactive Analysis (GEPIA) data set, and found significant downregulation of ZNF667-AS1 and ZNF667 in most of the tumor types, including bladder urothelial carcinoma (BLCA), breast invasive carcinoma (BRCA), and esophageal carcinoma (ESCA) (Fig. 1b; Supplementary Fig. S2). As shown in Fig. 1c, the relative expression levels of ZNF667-AS1 and ZNF667 in four esophageal cancer cells were significantly lower than that in normal esophageal epithelial cell line HEEpiC (*P* < 0.05). Similarly, the relative expression levels of ZNF667-AS1 and ZNF667 in ESCC tissues were significantly decreased compared with corresponding normal tissues (*P* < 0.05) (Fig. 1d). Among them, the expression levels of ZNF667-AS1 in 86 cases (63.7%) and ZNF667 in 78 cases (57.8%) of tumors were lower than that in the corresponding normal tissues by more than 50%. The expression levels of ZNF667-AS1 and ZNF667 were associated with TNM stage, pathological differentiation, lymph node metastasis, and distant metastasis or recurrence (Fig. 1e).

The expression level of ZNF667-AS1 was positively correlated with ZNF667 in ESCA in GEPIA data set (Fig. 1f). We also detected positive correlation between the expression level of ZNF667-AS1 and ZNF667 in ESCC tissues (*P* < 0.05) (Fig. 1g). The 5-year survival rate of ESCC patients with low ZNF667-AS1 or ZNF667 expression level (the expression level of ZNF667-AS1 or ZNF667 in tumor tissues was lower than 50% of that in the corresponding normal tissues) was significantly lower than that with high ZNF667-AS1 or ZNF667 expression level (*P* < 0.05) (Fig. 1h).

### Aberrant hypermethylation of CpG sites within proximal promoter of ZNF667-AS1 and ZNF667

**Fig. 2:**
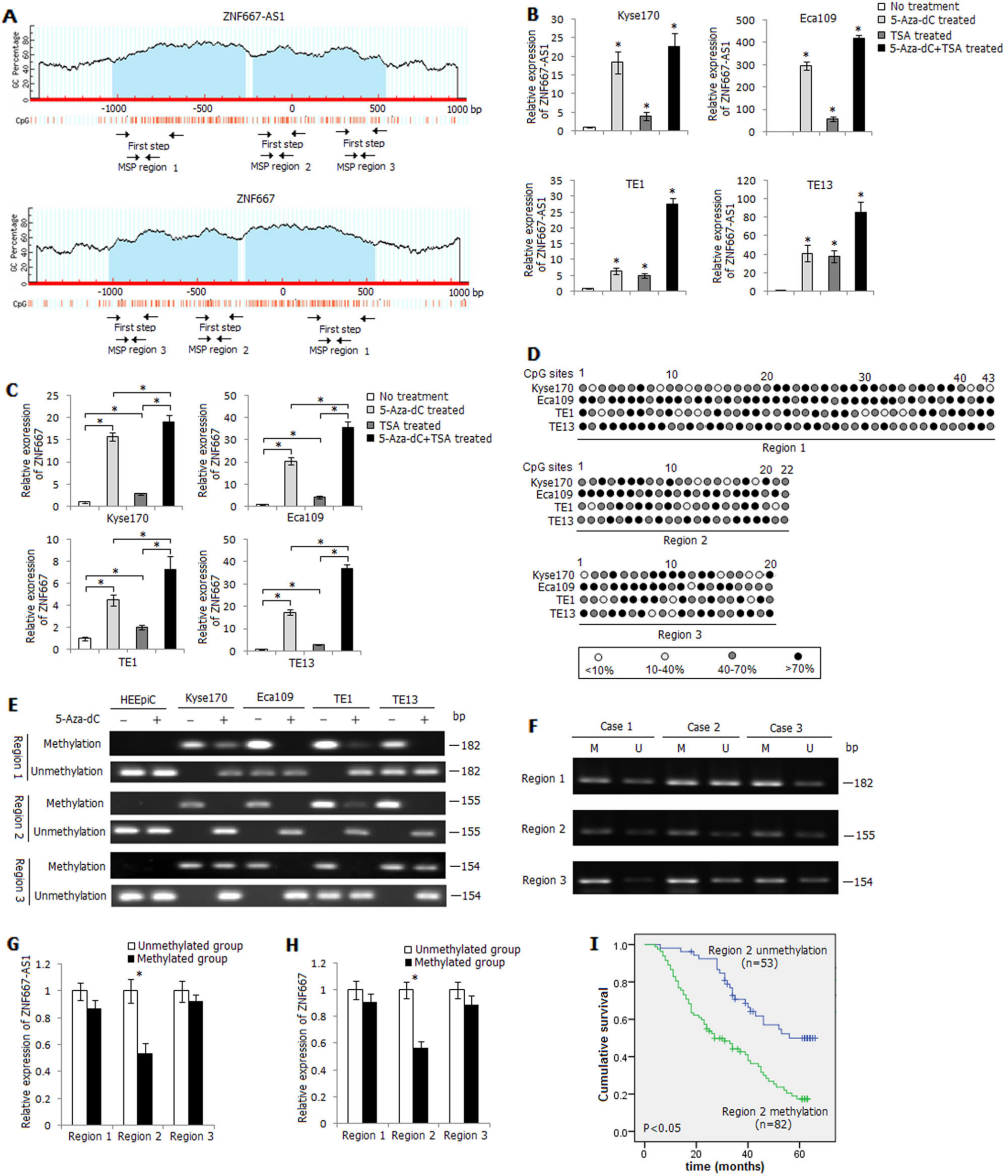
Methylation status of ZNF667-AS1 and ZNF667 in ESCC. **a** Schematic structure of ZNF667-AS1 and ZNF667 CpG islands predicted by MethPrimer. Three MSP regions analyzed are indicated. **b** Relative expression level of ZNF667-AS1 in 5-Aza-dC, TSA, or 5-Aza-dC+TSA treated esophageal cancer cells. **c** Relative expression level of ZNF667 in 5-Aza-dC, TSA, or 5-Aza-dC+TSA treated esophageal cancer cells. **d** High-resolution mapping of the methylation status of every CpG site in three regions by BGS assay in four esophageal cancer cell lines. Each CpG site is shown at the top row as an individual number. Percentage methylation was determined as percentage of methylated cytosines from 8 to 10 sequenced colonies. The color of circles for each CpG site represents the percentage of methylation. **e** Methylation status of three regions detected by BS-MSP assay in HEEpiC cells and esophageal cancer cells treated or untreated with 5-Aza-dC. **f** Methylation status of three regions detected by BS-MSP assay in ESCC tumor tissues. M: methylated; U: unmethylated. **g** Relative expression level of ZNF667-AS1 in tumor tissues with and without methylation of the three regions. **h** Relative expression level of ZNF667 in tumor tissues with and without methylation of the three regions. **i** The influence of region 2 methylation status on ESCC patients’ survival. *P < 0.05.

We next examined the role of DNA methylation in the inactivation of ZNF667-AS1 and ZNF667. We analyzed the distribution of CpG islands in the promoter and exon 1 regions of ZNF667-AS1 and ZNF667 by MethPrimer, and found two reversely distributed CpG islands spanning the promoter region to exon 1 of both genes (Fig. 2a). Four esophageal cancer cells were treated with DNA methyltransferase inhibitor 5-Aza-dC or histone deacetylase inhibitor TSA, as shown in Fig. 2b, c, the expression levels of ZNF667-AS1 and ZNF667 were significantly increased in the 5-Aza-dC or TSA treated cells, especially in the 5-Aza-dC+TSA treated cells.

The methylation status of CpG loci in the distal promoter, proximal promoter, and exon 1 regions of ZNF667-AS1 and ZNF667 was then verified by BGS assay, and frequent hypermethylation of CpG loci was observed in three regions (Fig. 2d). According to the distribution of methylated CpG loci in three regions detected by BGS assay, three pairs of BS-MSP primers were designed. As shown in Fig. 2e, hemimethylation or fully methylation of three regions was observed in esophageal cancer cells before 5-Aza-dC treatment, and fully methylation of region 2 was especially observed in four cancer cells. The methylation status of three regions was reversed after 5-Aza-dC treatment in four cancer cells. The methylation status of three regions in all tissue samples was further successfully examined by BS-MSP method (Fig. 2f). Of ESCC tissues and the corresponding normal tissues, methylation frequencies were as follows: 77.0% (104/135) and 34.1% (46/135) at region 1, 60.7% (82/135) and 11.1% (15/135) at region 2, 79.3% (107/135) and 20.7% (28/135) at region 3 (Supplementary Table 3). Methylation frequencies of three regions in ESCC tissues were significantly higher than those in corresponding normal tissues (*P* < 0.05). The methylation status of three regions in ESCC tissues was related to TNM stage and pathological differentiation (*P* < 0.05) (Supplementary Table 4). The expression of ZNF667-AS1 and ZNF667 was detected to be inversely correlated with the methylation status of region 2 (*P* < 0.05), but not with region 1 and region 3 (*P* > 0.05) (Fig. 2g, h). Furthermore, the 5-year survival rate of ESCC patients with region 2 hypermethylation was significantly lower compared with those with unmethylation of this region (*P* < 0.05) (Fig. 2i). Cox multivariate analysis further demonstrated that region 2 hypermethylation was also an independent prognostic factor for ESCC patients’ survival in addition to the factors such as TNM stage, pathological differentiation, lymph node metastasis, and family history of UGIC (Supplementary Table 5).

### In vitro proximal promoter methylation of ZNF667-AS1 and ZNF667 leads to a significant decrease in luciferase activity

**Fig. 3:**
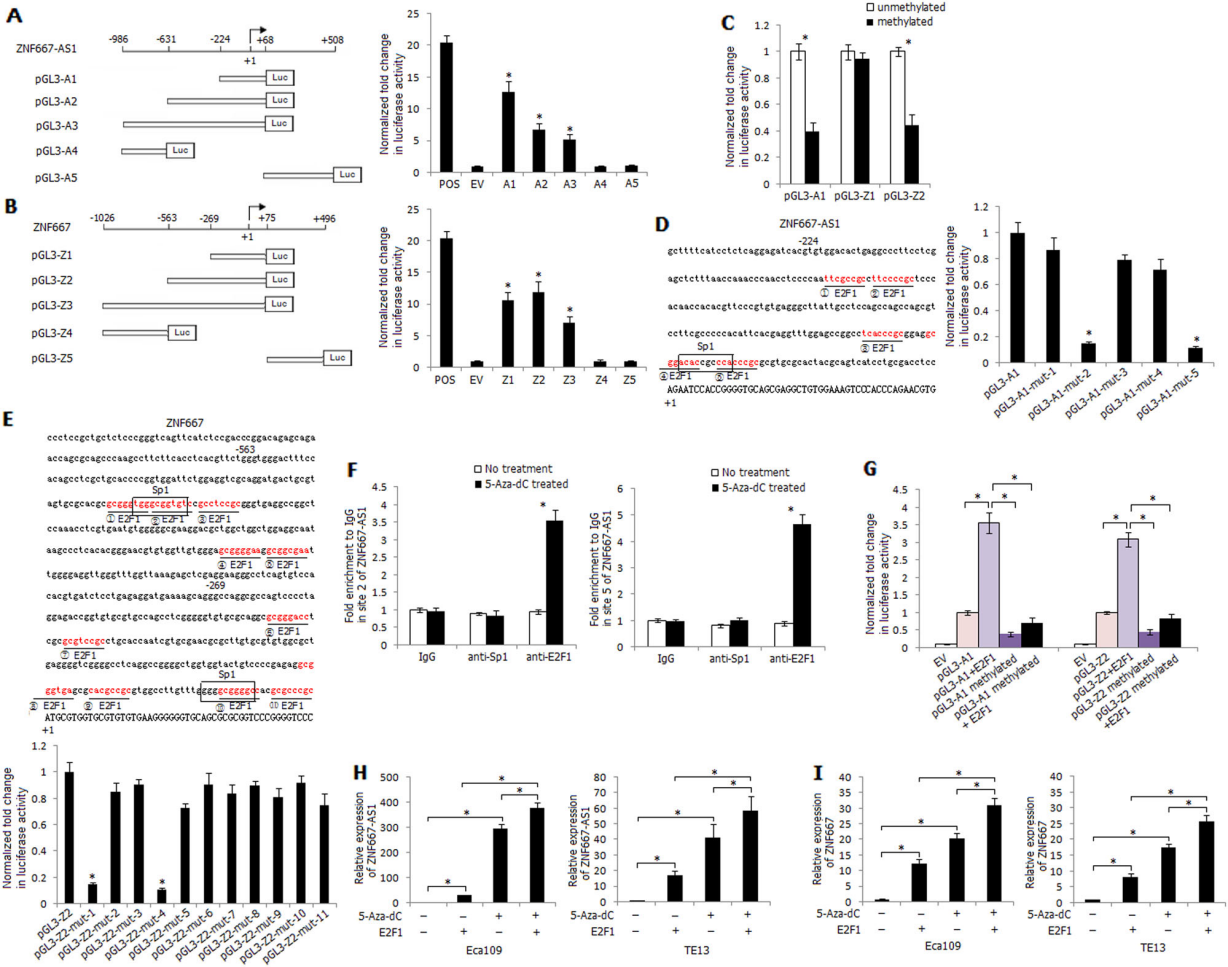
Effects of methylation and transcription factor E2F1 on ZNF667-AS1 and ZNF667 transcriptional activity. **a** Relative position and construction of reporter vectors of ZNF667-AS1. The pGL3-control vector was used as a positive control (POS), and empty pGL3-basic vector as a negative control (EV). **b** Relative position and construction of reporter vectors of ZNF667. POS: pGL3-control vector, EV: pGL3-basic vector. **c** Luciferase activity of unmethylated and methylated pGL3-A1, pGL3-Z1, and pGL3-Z2 reporter constructs in Eca109 cells. **d** Binding sites of transcription factor Sp1 and E2F1 in the promoter region of ZNF667-AS1, and luciferase activity of pGL3-A1 and pGL3-A1-mut (binding sites mutation) reporter constructs in Eca109 cells. **e** Binding sites of transcription factor Sp1 and E2F1 in the promoter region of ZNF667, and luciferase activity of pGL3-Z2 and pGL3-Z2-mut (binding sites mutation) reporter constructs in Eca109 cells. **f** Binding effect of site 2 or site 5 of ZNF667-AS1 with Sp1 or E2F1 in Eca109 cells. **g** Luciferase activity of different reporter constructs in Eca109 cells. *P < 0.05. **h** Relative expression of ZNF667-AS1 in 5-Aza-dC treated or E2F1 transfected Eca109 and TE13 cells. **i** Relative expression of ZNF667 in 5-Aza-dC treated or E2F1 transfected Eca109 and TE13 cells. The experiments are representative of three independent experiments with similar results. *P < 0.05.

We constructed reporter plasmids of ZNF667-AS1 and ZNF667 containing different portions of the unmethylated sequences (Fig. 3a, b). The pGL3-A1, pGL3-Z1, and pGL3-Z2 constructs demonstrated relative higher luciferase activity of all constructs detected by luciferase assay, suggesting the important role of proximal promoter methylation on transcriptional control of ZNF667-AS1 and ZNF667 (Fig. 3a, b). The luciferase activity of in vitro methylated pGL3-A1 and pGL3-Z2 was significantly decreased compared with unmethylated pGL3-A1 and pGL3-Z2 constructs, while methylated pGL3-Z1 construct demonstrated no apparent decrease of luciferase activity compared with unmethylated pGL3-Z1 construct (Fig. 3c). We further identified the key transcriptional factors that could regulate ZNF667-AS1 and ZNF667 transcription in ESCC cells. Using the online transcription factor prediction programmes, we found that the regions of pGL3-A1 and pGL3-Z2 constructs harbored crucial transcriptional regulatory elements, including potential Sp1- and E2F1-binding sites (Fig. 3d, e). Site-directed mutagenesis was used to abolish each binding site, and luciferase assays were performed. Compared with pGL3-A1, the luciferase activities of pGL3-A1-mut-2 and pGL3-A1-mut-5 constructs were significantly decreased by 85% and 88%, respectively; whereas the luciferase activities of pGL3-A1-mut-1, pGL3-A1-mut-3, and pGL3-A1-mut-4 were unvarying (Fig. 3d). Similarly, compared with pGL3-Z2, the luciferase activities of pGL3-Z2-mut-1 (corresponding to the sites of pGL3-A1-mut-5) and pGL3-Z2-mut-4 (corresponding to the sites of pGL3-A1-mut-2) were strikingly decreased among the eleven pGL3-Z2-mut constructs (Fig. 3e). It was suggested that the potential E2F1-binding sites at ZNF667-AS1 site 2 and site 5 (corresponding to ZNF667 site 4 and site 1) and the Sp1-binding site at ZNF667-AS1 site 5 (corresponding to ZNF667 site 1) were probably responsible for enhancing ZNF667-AS1 and ZNF667 transcription. The binding properties of E2F1 or Sp1 at site 2 or site 5 of ZNF667-AS1 were further validated by ChIP assay in Eca109 cells with or without 5-Aza-dC treatment. As shown in Fig. 3f, significant increased enrichment in site 2 and site 5 with anti-E2F1 antibody was observed in 5-Aza-dC treated Eca109 cells compared with control, while no apparent variation was detected with anti-Sp1 antibody in 5-Aza-dC treated Eca109 cells, suggesting the key transcriptional regulation of E2F1 on ZNF667-AS1 and ZNF667 transcription within the proximal promoter.

Co-transfection of pGL3-A1 or pGL3-Z2 and E2F1 significantly raised the luciferase activity, while the stimulating effect was abrogated when pGL3-A1 or pGL3-Z2 construct was in vitro methylated, suggesting that CpG sites hypermethylation within proximal promoter of ZNF667-AS1 or ZNF667 might abolish E2F1 binding and the following transcriptional activation of them (Fig. 3g). Furthermore, 5-Aza-dC treatment and E2F1 overexpression significantly increased the expression level of ZNF667-AS1 and ZNF667 compared with single 5-Aza-dC treatment or E2F1 overexpression cells (Fig. 3h, i).

### ZNF667-AS1 and ZNF667 inhibit esophageal cancer cells viability, migration, and invasion in vitro

**Fig. 4:**
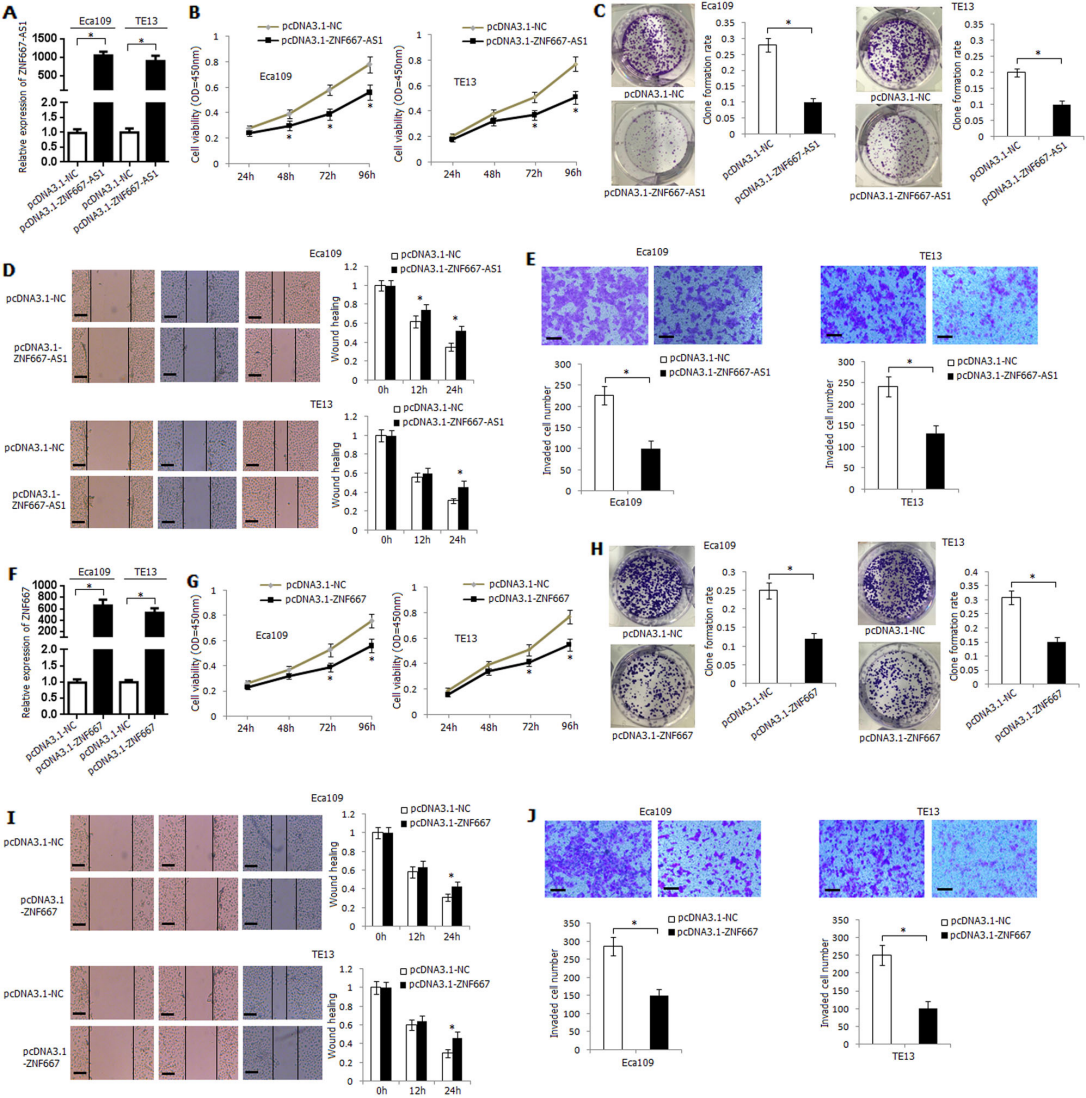
Functional analysis of ZNF667-AS1 and ZNF667 in human esophageal cancer cell lines. **a** Transfection efficiency and ZNF667-AS1 expression level in pcDNA3.1-ZNF667-AS1 transfected Eca109 and TE13 cells. **b** The influence of overexpression of ZNF667-AS1 on Eca109 and TE13 cells viability. **c** The influence of overexpression of ZNF667-AS1 on Eca109 and TE13 cells clone-formation rate. **d** The influence of overexpression of ZNF667-AS1 on Eca109 and TE13 cells migration ability. **e** The influence of overexpression of ZNF667-AS1 on Eca109 and TE13 cells invasion ability. **f** Transfection efficiency and ZNF667 expression level in pcDNA3.1-ZNF667-transfected Eca109 and TE13 cells. **g** The influence of overexpression of ZNF667 on Eca109 and TE13 cells viability. **h** The influence of overexpression of ZNF667 on Eca109 and TE13 cells clone-formation rate. **i** The influence of overexpression of ZNF667 on Eca109 and TE13 cells migration ability. **j** The influence of overexpression of ZNF667 on Eca109 and TE13 cells invasion ability. For all quantitative results, the data are presented as the mean ± SD. The experiments are representative of three independent experiments with similar results. *P < 0.05.

The construct containing ZNF667-AS1 transcripts (pcDNA3.1-ZNF667-AS1) was transfected into Eca109 and TE13 cells. The expression level of ZNF667-AS1 in transfected Eca109 and TE13 cells was strikingly upregulated (Fig. 4a). Overexpression of ZNF667-AS1 significantly inhibited the viability, clone-formation rate, migration ability, and invasive ability of Eca109 and TE13 cells (Fig. 4b–e). We further knocked down the expression of ZNF667-AS1 using short hairpin RNAs (shRNAs) in Kyse170 cells, and shRNA-ZNF667-AS1-1 demonstrated the strongest knockdown efficiency (Supplementary Fig. 3A). Knockdown of ZNF667-AS1 significantly increased the viability, migration, and invasion ability of Kyse170 cells (Supplementary Fig. 3B–D).

ZNF667 overexpression plasmid (pcDNA3.1-ZNF667) was transfected into Eca109 and TE13 cells, and the expression level of ZNF667 was significantly upregulated (Fig. 4f). Overexpression of ZNF667 significantly inhibited the viability and clone-formation rate of Eca109 and TE13 cells (Fig. 4g, h). Moreover, pcDNA3.1-ZNF667 transfection significantly inhibited migration and invasive ability of Eca109 and TE13 cells (Fig. 4i, j). The expression of ZNF667 was also knocked down using shRNAs in Kyse170 cells (Supplementary Fig. 3E), and knockdown of ZNF667 significantly increased the viability, migration, and invasion ability of Kyse170 cells (Supplementary Fig. 3F–H).

### Overexpression of ZNF667-AS1 increases mRNA and protein expression level of ZNF667

**Fig. 5:**
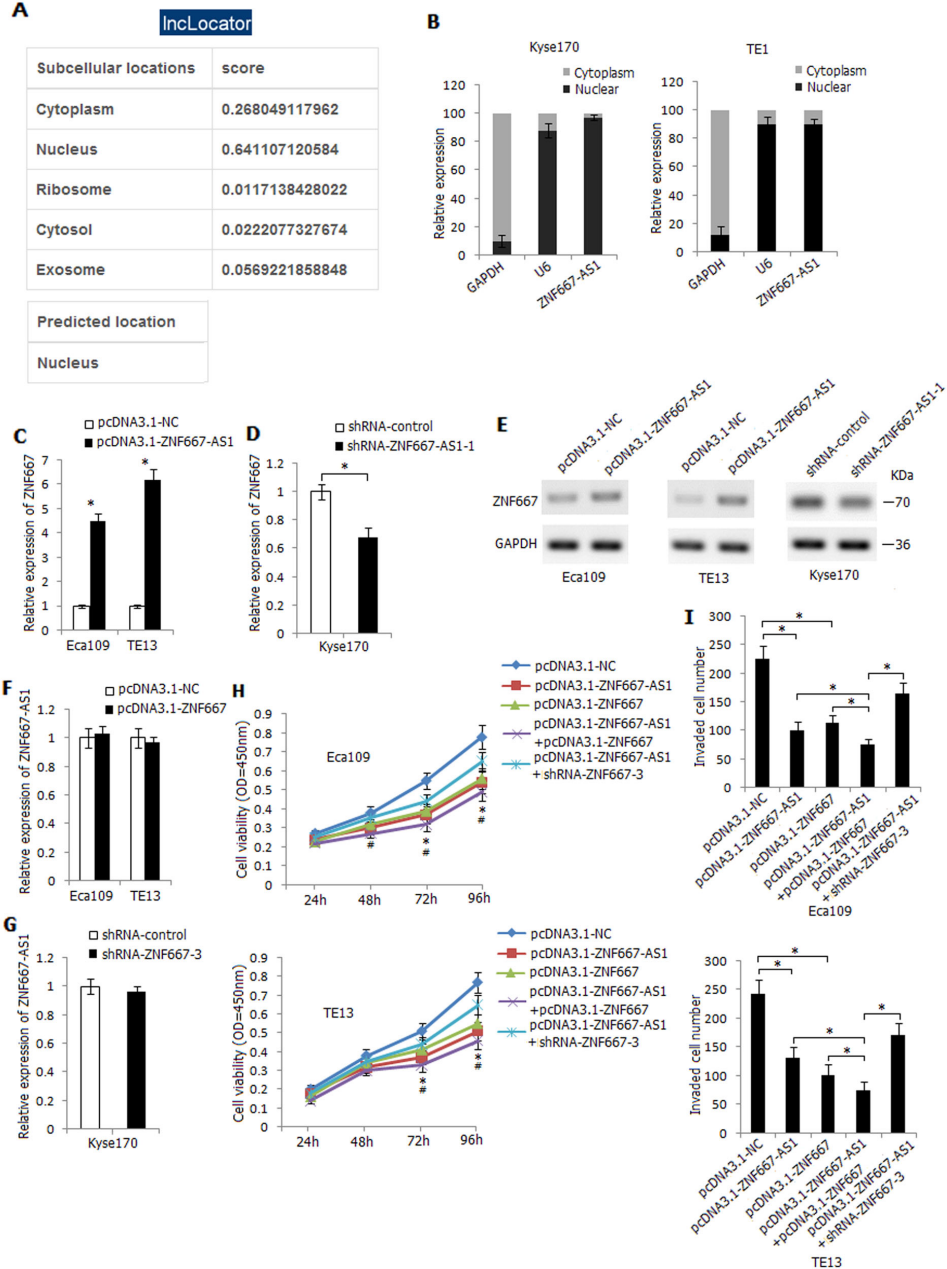
Regulatory effect between ZNF667-AS1 and ZNF667 in human esophageal cancer cell lines. **a** Subcellular localization of ZNF667-AS1 predicted by lncLocator (http://www.csbio.sjtu.edu.cn/bioinf/lncLocator/). **b** The expression level of ZNF667-AS1 in nuclear and cytoplasmic fractions of Kyse170 and TE1 cells detected by qRT-PCR. GAPDH: cytoplasmic control; U6: nuclear control. **c** The influence of overexpression of ZNF667-AS1 on mRNA expression level of ZNF667 in Eca109 and TE13 cells. **d** The influence of knockdown of ZNF667-AS1 on mRNA expression level of ZNF667 in Kyse170 cells. **e** The influence of overexpression or knockdown of ZNF667-AS1 on protein expression level of ZNF667. **f** The influence of overexpression of ZNF667 on expression level of ZNF667-AS1 in Eca109 and TE13 cells. **g** The influence of knockdown of ZNF667 on expression level of ZNF667-AS1 in Kyse170 cells. **h** The combined effect of ZNF667-AS1 and ZNF667 on Eca109 and TE13 cells viability. *P < 0.05, pcDNA3.1-ZNF667-AS1 + pcDNA3.1-ZNF667 compared with pcDNA3.1-ZNF667-AS1 or pcDNA3.1-ZNF667, ^#^*P* < 0.05, pcDNA3.1-ZNF667-AS1 + shRNA-ZNF667-3 compared with pcDNA3.1-ZNF667-AS1 + pcDNA3.1-ZNF667. **i** The combined effect of ZNF667-AS1 and ZNF667 on Eca109 and TE13 cells invasive ability. The experiments are representative of three independent experiments with similar results. *P < 0.05.

It has been reported that antisense lncRNAs could regulate the expression and function of sense transcript^18,19^. ZNF667-AS1 was mainly located in the nucleus of cells predicted by lncLocator (Fig. 5a), and it was detected to be mainly distributed in the nucleus of Kyse170 and TE1 cells (Fig. 5b). Overexpression of ZNF667-AS1 led to an increased mRNA expression level of ZNF667 in Eca109 and TE13 cells (Fig. 5c), conversely, knockdown of ZNF667-AS1 decreased mRNA expression level of ZNF667 in Kyse170 cells (Fig. 5d). Similarly, overexpression or knockdown of ZNF667-AS1 led to an increased or reduced protein expression level of ZNF667 (Fig. 5e). However, overexpression or knockdown of ZNF667 could not affect the expression level of ZNF667-AS1 in esophageal cancer cells (Fig. 5f, g). Co-transfection of pcDNA3.1-ZNF667-AS1 and pcDNA3.1-ZNF667 significantly decreased Eca109 and TE13 cells viability and invasiveness compared with solely pcDNA3.1-ZNF667-AS1 or pcDNA3.1-ZNF667 transfected cells, moreover, co-transfection of pcDNA3.1-ZNF667-AS1 and shRNA-ZNF667-3 partially reversed the intense reduction effect, indicating the synergistic effects of ZNF667-AS1 and ZNF667 on inhibiting esophageal cancer cells viability and invasion (Fig. 5h, i).

### ZNF667-AS1 interacts with and recruits TET1 to its target gene ZNF667 and E-cadherin to activate their expression

**Fig. 6:**
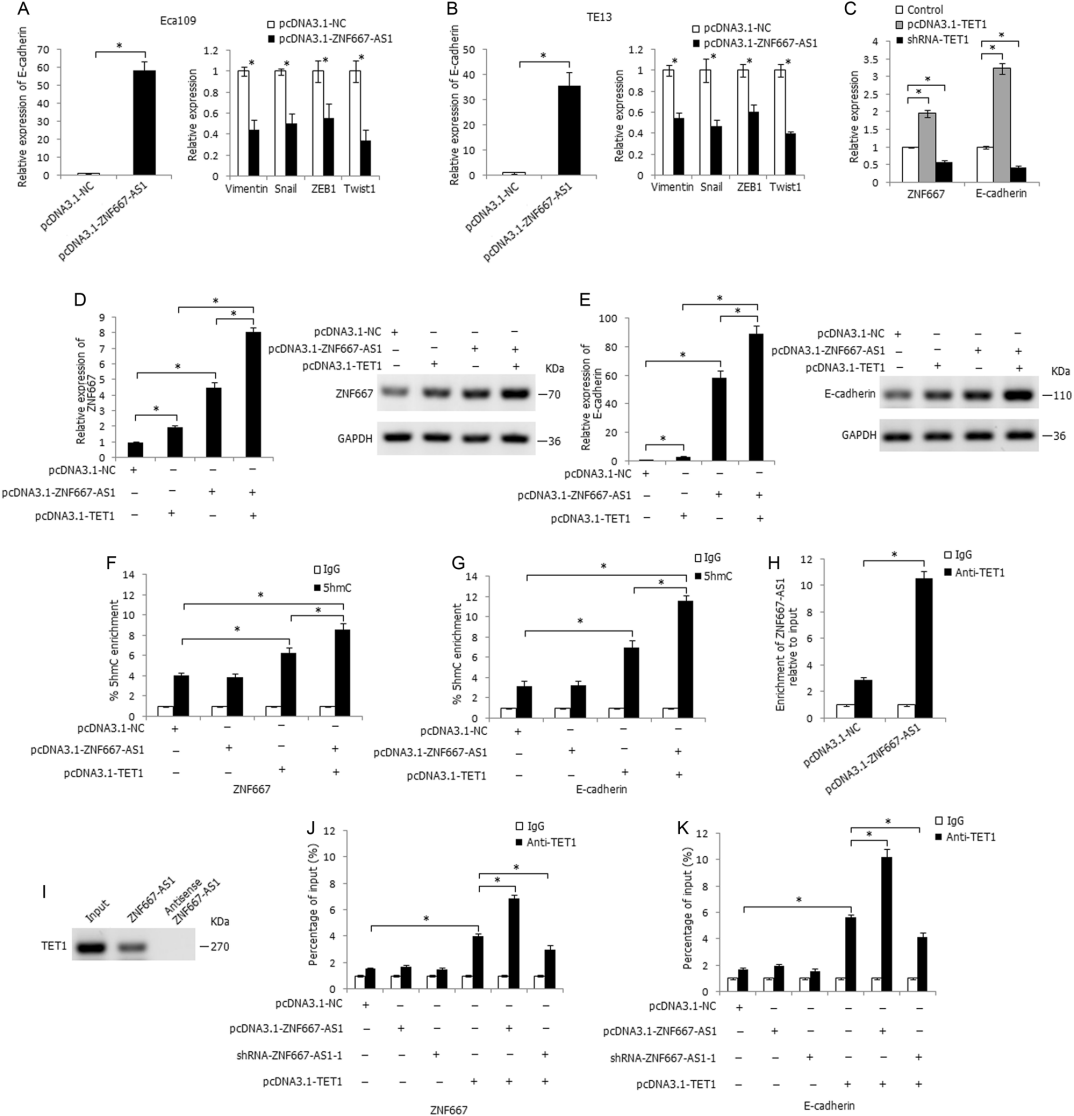
ZNF667-AS1 regulates the expression of ZNF667 and E-cadherin through interacting with TET1. **a** The influence of overexpression of ZNF667-AS1 on expression level of E-cadherin, Vimentin, Snail, ZEB1, and Twist1 in Eca109 cells. **b** The influence of overexpression of ZNF667-AS1 on expression level of E-cadherin, Vimentin, Snail, ZEB1, and Twist1 in TE13 cells. **c** The influence of overexpression or knockdown of TET1 on expression level of ZNF667 and E-cadherin. **d** The co-regulatory effect of ZNF667-AS1 and TET1 on mRNA and protein expression level of ZNF667 in Eca109 cells. **e** The co-regulatory effect of ZNF667-AS1 and TET1 on mRNA and protein expression level of E-cadherin in Eca109 cells. **f** The 5hmC enrichment at the promoter region of ZNF667 in Eca109 cells, determined by hMeDIP-qPCR method. **g** The 5hmC enrichment at the promoter region of E-cadherin in Eca109 cells, determined by hMeDIP-qPCR method. **h** The binding effect of ZNF667-AS1 with TET1 in ZNF667-AS1 transfected Eca109 cells, determined by RIP assay. **i** The binding effect of ZNF667-AS1 with TET1, determined by RNA pull-down assay. **j** The co-regulation of ZNF667-AS1 and TET1 on enrichment of TET1 at the promoter region of ZNF667, determined by ChIP assay. **k** The co-regulation of ZNF667-AS1 and TET1 on enrichment of TET1 at the promoter region of E-cadherin, determined by ChIP assay. *P < 0.05.

For the inhibitory effect of ZNF667-AS1 on esophageal cancer cells migration and invasion, we further investigated the role of ZNF667-AS1 in epithelial–mesenchymal transition (EMT) process. As shown in Fig. 6a, b, overexpression of ZNF667-AS1 in Eca109 and TE13 cells significantly increased the mRNA expression level of the epithelial marker E-cadherin, and decreased the mRNA expression level of the mesenchymal markers and transcription factors Vimentin, Snail, ZEB1, and Twist1.

For the more evident expression changes of E-cadherin in ZNF667-AS1-transfected cells, we further focused on the regulatory effect of ZNF667-AS1 on E-cadherin. Due to the nucleus distribution of ZNF667-AS1, we considered the regulatory role of ZNF667-AS1 at the genomic level. Hypermethylation of E-cadherin at promoter CpG sites is a recognized mechanism of its inactivation in many cancers. Recent studies have shown that ten–eleven translocation (TET) gene family (including TET1, TET2, and TET3) can hydrolyze 5′-methylcytosine (5′-mc) to 5′-hydroxymethylcytosine (5′-hmc), and eventually remove methyl from the CpG dinucleotide to activate gene expression^20^. E-cadherin is the reported TET target gene^21,22^; ZNF667 could be regulated by promoter CpG sites methylation, so we considered whether ZNF667-AS1 interacts with TET to regulate the expression of E-cadherin and ZNF667. We predicted the binding ability of ZNF667-AS1 with TET1, TET2, and TET3 by RPISeq and found the greatest interaction probability between TET1 protein and ZNF667-AS1 (RF: 0.75 and SVM: 0.99). Overexpression of TET1 significantly increased the expression level of ZNF667 and E-cadherin in Eca109 cells, while knockdown of TET1 demonstrated the opposite effect (Fig. 6c). As shown in Fig. 6d, co-transfection of pcDNA3.1-ZNF667-AS1 and pcDNA3.1-TET1 significantly enhanced the mRNA and protein expression level of ZNF667 compared to solely pcDNA3.1-ZNF667-AS1 or pcDNA3.1-TET1 transfected groups, similarly, co-transfection of pcDNA3.1-ZNF667-AS1 and pcDNA3.1-TET1 strikingly increased the mRNA and protein expression level of E-cadherin (Fig. 6e). The hMeDIP-qPCR assay was further used to track the 5hmC change in the CpG-rich regions of ZNF667 and E-cadherin promoters, and co-transfection of pcDNA3.1-ZNF667-AS1 and pcDNA3.1-TET1 in Eca109 cells significantly increased 5hmC levels at the promoter regions of ZNF667 and E-cadherin (Fig. 6f, g). Notably, RIP assay demonstrated the binding of ZNF667-AS1 with TET1 protein in ZNF667-AS1 transfected Eca109 cells (Fig. 6h), and the results were further verified by RNA pull-down assay (Fig. 6i). ChIP analysis further demonstrated the binding of TET1 to the promoter regions of ZNF667 and E-cadherin, and overexpression of ZNF667-AS1 significantly increased the enrichment of TET1 at the promoter regions of ZNF667 and E-cadherin, whereas knockdown of ZNF667-AS1 partially attenuated the enrichment of TET1 (Fig. 6j, k). These results demonstrated that ZNF667-AS1 could interact with and recruit TET1 to its target gene ZNF667 and E-cadherin to hydrolyze 5′-mc to 5′-hmc and further activate their expression.

### ZNF667-AS1 interacts with UTX to decrease histone H3K27 tri-methylation to activate ZNF667 and E-cadherin expression

**Fig. 7:**
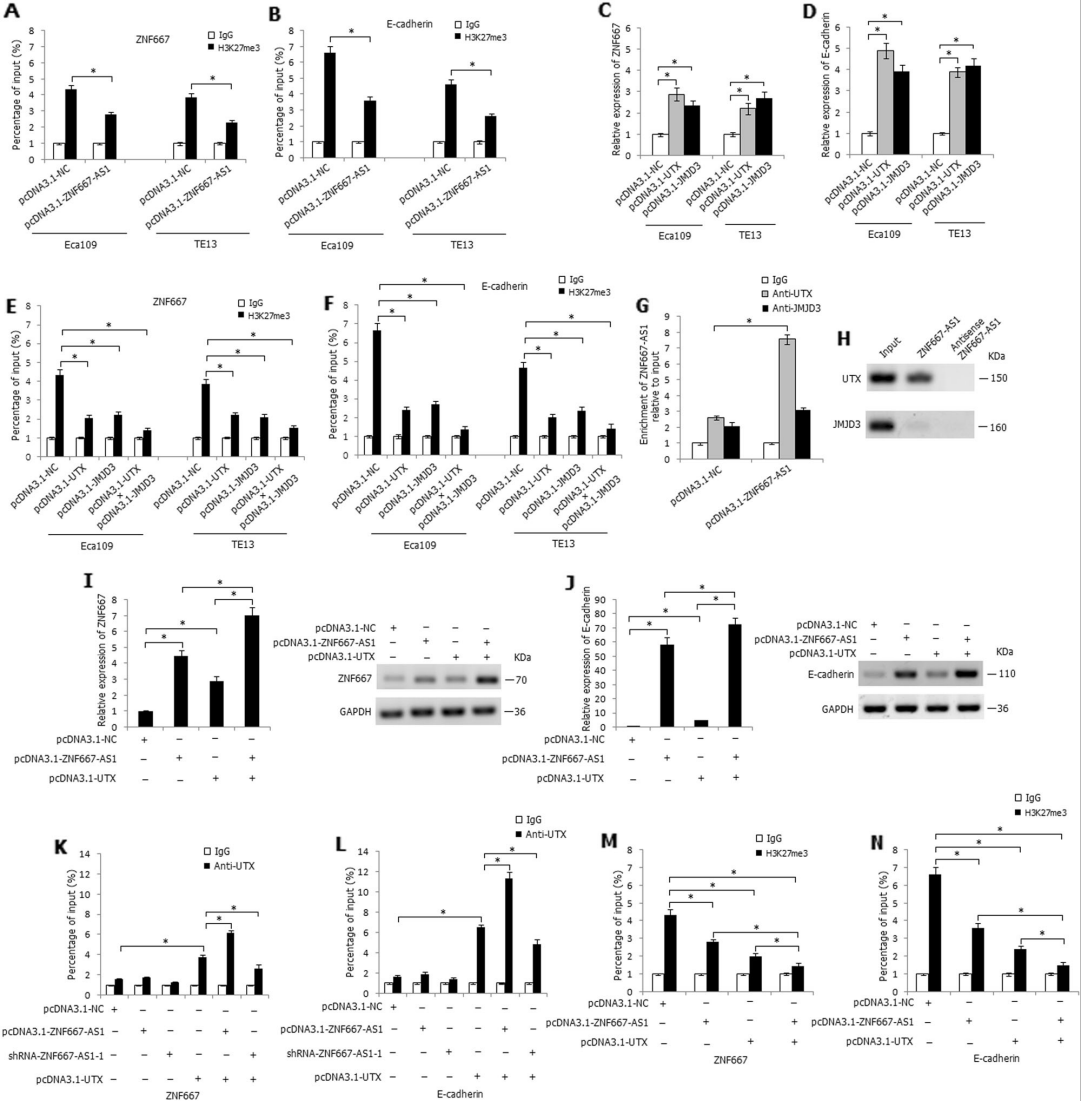
ZNF667-AS1 regulates the expression of ZNF667 and E-cadherin through interacting with UTX. **a** The regulation of ZNF667-AS1 on enrichment of H3K27me3 at the promoter region of ZNF667, determined by ChIP assay. **b** The regulation of ZNF667-AS1 on enrichment of H3K27me3 at the promoter region of E-cadherin, determined by ChIP assay. **c** The influence of overexpression of UTX or JMJD3 on expression level of ZNF667. **d** The influence of overexpression of UTX or JMJD3 on expression level of E-cadherin. **e** The regulation of UTX or JMJD3 on enrichment of H3K27me3 at the promoter region of ZNF667, determined by ChIP assay. **f** The regulation of UTX or JMJD3 on enrichment of H3K27me3 at the promoter region of E-cadherin, determined by ChIP assay. **g** The binding effect of ZNF667-AS1 with UTX in ZNF667-AS1 transfected Eca109 cells, determined by RIP assay. **h** The binding effect of ZNF667-AS1 with UTX, determined by RNA pull-down assay. **i** The co-regulatory effect of ZNF667-AS1 and UTX on mRNA and protein expression level of ZNF667 in Eca109 cells. **j** The co-regulatory effect of ZNF667-AS1 and UTX on mRNA and protein expression level of E-cadherin in Eca109 cells. **k** The enrichment of UTX at the promoter region of ZNF667, determined by ChIP assay. **l** The enrichment of UTX at the promoter region of E-cadherin, determined by ChIP assay. **m** The enrichment of H3K27me3 at the promoter region of ZNF667, determined by ChIP assay. **n** The enrichment of H3K27me3 at the promoter region of E-cadherin, determined by ChIP assay. *P < 0.05.

TET1 mediates a cross talk between DNA methylation and histone modifications to orchestrate transcriptional silencing^23^. Loss of TET1 increased EZH2 expression and reduced UTX expression in DLD1 cells, thus increasing histone H3K27 tri-methylation on target gene promoter and leading to repression of the target gene^21^. We predicted the binding ability of ZNF667-AS1 with histone lysine demethylase UTX and JMJD3 by RPISeq and found the greatest interaction probability between ZNF667-AS1 and UTX protein (RF: 0.8 and SVM: 0.99), and JMJD3 protein (RF: 0.85 and SVM: 0.98). As shown in Fig. 7a, b, overexpression of ZNF667-AS1 decreased the enrichment of H3K27me3 at the promoter region of ZNF667 and E-cadherin in Eca109 and TE13 cells, determined by ChIP assay. We further constructed UTX and JMJD3 overexpression plasmids and, respectively, transfected into Eca109 and TE13 cells, overexpression of UTX or JMJD3 significantly enhanced the mRNA expression level of ZNF667 and E-cadherin (Fig. 7c, d). Furthermore, overexpression of UTX or JMJD3 significantly decreased the enrichment of H3K27me3 at the promoter region of ZNF667 and E-cadherin, and co-overexpression of UTX and JMJD3 demonstrated the strongest effect (Fig. 7e, f). RIP and RNA pull-down assays verified the binding effect of ZNF667-AS1 and UTX (Fig. 7g, h). As shown in Fig. 7i, j, co-overexpression of ZNF667-AS1 and UTX strikingly increased the mRNA and protein expression level of ZNF667 and E-cadherin in Eca109 cells, compared with the solely pcDNA3.1-ZNF667-AS1 or pcDNA3.1-UTX transfected cells. ChIP assay further demonstrated that overexpression of ZNF667-AS1 increased the enrichment of UTX at the promoter region of ZNF667 and E-cadherin in Eca109 cells, whereas knockdown of ZNF667-AS1 partially attenuated the enrichment of UTX (Fig. 7k, l). Moreover, co-overexpression of ZNF667-AS1 and UTX strikingly decreased the enrichment of H3K27me3 at the promoter region of ZNF667 and E-cadherin, determined by ChIP assay (Fig. 7m, n).

### TET1 and UTX inhibit the viability and invasion of esophageal cancer cells

**Fig. 8:**
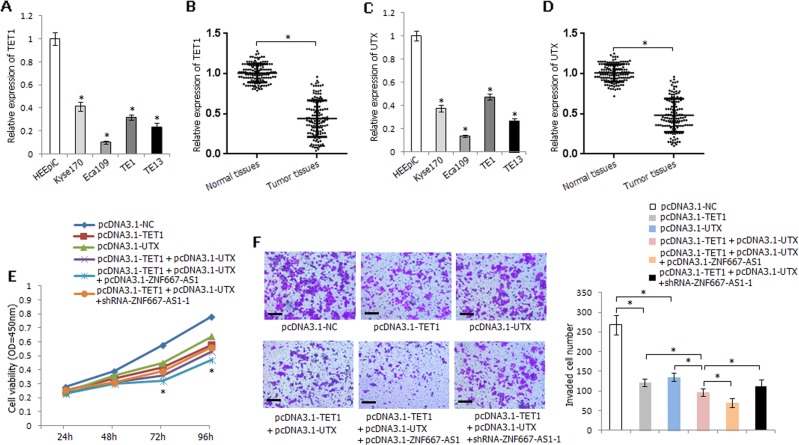
Expression level and functional analyses of TET1 and UTX in ESCC. **a** Relative expression level of TET1 in HEEpiC and four esophageal cancer cell lines. **b** Relative expression level of TET1 in ESCC tissues and corresponding normal tissues. **c** Relative expression level of UTX in HEEpiC and four esophageal cancer cell lines. **d** Relative expression level of UTX in ESCC tissues and corresponding normal tissues. **e** The influence of TET1, UTX, and ZNF667-AS1 on Eca109 cells viability. *P < 0.05, pcDNA3.1-TET1 or pcDNA3.1-UTX compared with pcDNA3.1-NC; pcDNA3.1-TET1 + pcDNA3.1-UTX compared with pcDNA3.1-TET1 or pcDNA3.1-UTX; pcDNA3.1-TET1 + pcDNA3.1-UTX + pcDNA3.1-ZNF667-AS1 compared with pcDNA3.1-TET1 or pcDNA3.1-UTX or pcDNA3.1-TET1 + pcDNA3.1-UTX; pcDNA3.1-TET1 + pcDNA3.1-UTX + shRNA-ZNF667-AS1-1 compared with pcDNA3.1-TET1 + pcDNA3.1-UTX + pcDNA3.1-ZNF667-AS1. **f** The influence of TET1, UTX, and ZNF667-AS1 on Eca109 cells invasive ability. The experiments are representative of three independent experiments with similar results. *P < 0.05.

The mRNA expression level of TET1 was significantly decreased in esophageal cancer cells and ESCC tissues (Fig. 8a, b); UTX demonstrated the similar expression level in esophageal cancer cells and ESCC tissues (Fig. 8c, d). Single transfection of pcDNA3.1-TET1 or pcDNA3.1-UTX decreased the viability of Eca109 cells, while co-transfection of pcDNA3.1-TET1 and pcDNA3.1-UTX significantly decreased the viability of Eca109 cells than the solely transfected cells; furthermore, co-overexpression of ZNF667-AS1, TET1, and UTX strikingly inhibited the viability of Eca109 cells, and co-overexpression of TET1 and UTX accompanied with knockdown of ZNF667-AS1 partially offseted the inhibitory effect (Fig. 8e). Similarly, co-overexpression of ZNF667-AS1, TET1, and UTX strikingly inhibited the invasive ability of Eca109 cells, while co-overexpression of TET1 and UTX accompanied with knockdown of ZNF667-AS1 partially reversed the inhibitory effect (Fig. 8f).

## Discussion

As a head-to-head lncRNA, ZNF667-AS1 and its antisense transcript ZNF667 are both located in 19q13.43. The role of ZNF667-AS1 and ZNF667 has been reported in several types of cancers^11–14,16,24^; however, the functional role of them in ESCC has not been clarified. ZNF667-AS1 was first reported in a study using microarray analysis to analyze the changed genes in immortalized human mammary epithelial cells (HMEC), and ZNF667-AS1 was found to be expressed in all normal finite lifespan human cells examined to date, and was downregulated or inactivated in immortalized HMEC^11^. ZNF667-AS1 silencing during immortalization was linked to the aberrant epigenetic event of DNA hypermethylation of its CpG island promoter. This epigenetic silencing was also seen in human breast cancer cell lines and in a majority of human breast tumor tissues. Furthermore, analysis of TCGA data across 16 human cancers revealed that deregulation of ZNF667-AS1 expression due to DNA hypermethylation was a frequent event in most common human cancers^11^. After that, another study evaluated the epigenetic and transcriptional state of ZNF667-AS1 in two premalignant conditions—ductal carcimomas in situ and colon adenomas. The results showed that ZNF667-AS1 silencing was an early epigenetic event in human carcinogenesis, and this epigenetic silencing was maintained throughout malignant transformation and metastatic growth^12^. ZNF667-AS1 was also found to be downregulated in cervical cancer tissues^13^. Similarly, Aberrant methylation and downregulation of ZNF667-AS1 promoted the malignant progression of laryngeal squamous cell carcinoma^16^. In this study, we also found downregulation of ZNF667-AS1 in esophageal cancer cell lines and ESCC tissues, and proximal promoter hypermethylation may be one of the mechanisms in leading to its silencing; together with the inhibiting effect of ZNF667-AS1 on esophageal cancer cells viability, migration, and invasion; suggesting the tumor-suppressor role of ZNF667-AS1 in ESCC tumorigenesis.

ZNF667 is a novel zinc finger protein, which was found to be upregulated by myocardial ischemic preconditioning (IPC); and it played a neuroprotective role by acting as a transcription factor in cerebral IPC^25^. ZNF667 was also detected to inhibit the expression and the promoter activity of the rat proapoptotic gene Bax gene, and at the same time prevented apoptosis of H9c2 cells, induced by H2O2 and Dox^26^. Vrba group detected downregulation of ZNF667 during immortalization, although much less dramatically than ZNF667-AS1^11^. ZNF667 was also found to be downregulated in laryngeal squamous cell carcinoma tissues^16^. We also detected downregulation of ZNF667 in esophageal cancer cells and ESCC tissues, and overexpression of ZNF667 could inhibit the viability, migration, and invasion of esophageal cancer cells, suggesting that ZNF667 may act as tumor-suppressor gene in ESCC occurrence and development.

DNA methylation has been reported to regulate the expression of protein-coding genes and lncRNAs^27^. The ZNF667-AS1 promoter overlaps a large CpG island that is shared with ZNF667. We found intimate correlation between proximal promoter methylation and their expression, suggesting the important role of proximal promoter methylation on their inactivation. The promoter region of gene contains many binding sites of transcription factors and aberrant CpG dinucleotides hypermethylation in this region may directly or indirectly affect the binding ability of transcription factors. Further analyses verified the CpG sites hypermethylation within two binding sites of E2F1 in the proximal promoter region of ZNF667-AS1 and ZNF667 may influence the binding ability of E2F1 to the binding sites, and further led to the transcriptional inhibition of ZNF667-AS1 and ZNF667. Moreover, pGL3-Z1 construct (−269 to +75 bp) also demonstrated relative higher luciferase activity, however, methylated pGL3-Z1 construct demonstrated no apparent decrease of luciferase activity compared with unmethylated pGL3-Z1 construct, suggesting that other transcription factors may also participate in the transcriptional regulation of ZNF667 and the binding ability cannot be influenced by CpG dinucleotides hypermethylation. Further studies are worth doing to warrant the hypothesis.

It has been reported that antisense lncRNAs can regulate the expression of sense transcripts in cis, and lncRNAs located in the nucleus may guide and recruit transcription factors or DNA or histone protein modification enzymes to specific genomic loci to regulate gene expression^28^. ZNF667-AS1 was mainly located in the nucleus of esophageal cancer cells, and could upregulate the mRNA and protein expression of ZNF667. Furthermore, ZNF667-AS1 could strikingly increase the expression of E-cadherin. As an epithelial marker, E-cadherin was inactivated in many kinds of cancers, and its inactivation was partly attributed to the aberrant promoter CpG sites hypermethylation^29,30^. The methylation of cytosine was for a long time assumed to be permanent, but the discovery of the ability of the TET enzymes to convert 5mC to 5hmC, and further to 5fC and 5caC has suggested a new mechanism by which DNA can be demethylated. E-cadherin has been reported to be the TET target gene^21,22^, and ZNF667 could be regulated by CpG sites methylation. In this study, promoter CpG sites hypermethylation status of ZNF667 and E-cadherin was found to be demethylated by TET1, and ZNF667-AS1 could interact and recruit TET1 to ZNF667 and E-cadherin to hydrolyze 5′-mc to 5′-hmc and further activates their expression. Furthermore, ZNF667-AS1 could also interact with UTX to decrease histone H3K27 tri-methylation to activate ZNF667 and E-cadherin expression. Thus, ZNF667-AS1 may regulate the transcription and expression of ZNF667 and E-cadherin by interacting with TET1 and UTX to change DNA and H3K27 methylation status within the promoter region of them.

In all, ZNF667-AS1 and its sense transcript ZNF667 may act as tumor-suppressor genes in ESCC, and both inhibit esophageal cancer cells viability, migration, and invasion. The expression of ZNF667-AS1 and ZNF667 is simultaneously regulated by proximal promoter methylation, and ZNF667-AS1 regulates the expression of ZNF667 and E-cadherin by interacting with TET1 and UTX. Furthermore, ZNF667-AS1 and ZNF667 may serve as potential prognostic markers in predicting ESCC patients’ survival.

## Materials and methods

### Patients and specimens

A total of 135 ESCC patients who received surgery at the Fourth Affiliated Hospital of Hebei Medical University from 2008 and 2013 were considered in this study. The patients included 99 males and 36 females, with a median age of 59.3 years (ranged 39–73 years). Written informed consent was obtained from all patients, who had not received radiotherapy, chemotherapy, or biotherapy before operation. Primary ESCC tissues and adjacent corresponding normal tissues were obtained from all ESCC cases. The tissue specimens were divided into two parallel parts: one part was frozen and preserved at −80 °C to extract genomic DNA and RNA; the other part was fixed in formalin and embedded in paraffin. Information on clinical data and clinicopathological features was obtained from hospital records and pathological diagnosis. Individuals with at least one first-degree relative or at least 2 s-degree relatives with esophageal/cardia/gastric cancer are defined as having a family history of upper gastrointestinal cancer (UGIC). Recurrence and survival data were obtained from the tumor registry and hospital chart review (Supplementary Table 1). The study was approved by the Ethics Committee of the Fourth Affiliated Hospital, Hebei Medical University.

### Cell lines and treatment

Human esophageal cancer cell lines (Kyse170, Eca109, TE1, and TE13) were cultured in the RPMI-1640 medium (Invitrogen, Carlsbad, CA, USA), supplemented with 10% heat-inactivated fetal bovine serum (FBS) (Invitrogen, Carlsbad, CA, USA) at 37 °C and 5% CO_2_. Human normal esophageal epithelial cell line HEEpiC was cultured according to the manufacturer’s instructions. When needed, cells (2 × 10^5^/mL) were treated with 5 μM DNA methyltransferase inhibitor 5-aza-2′-deoxycytidine (5-Aza-dC) (Sigma, St Louis, MO, USA) for 72 h with 5-Aza-dC replenishment every 24 h; or with 0.3 μM histone deacetylase inhibitor TSA (Sigma, St Louis, MO, USA) for 24 h; or with the combination of 5 μM 5-Aza-dC for 48 h followed by 0.3 μM TSA for an additional 24 h. Control cells received no drug treatment. The dose and time of 5-Aza-dC and TSA were based on preliminary studies, which showed the optimal gene reactivation^31^.

### RNA extraction and quantitative real-time RT-PCR (qRT-PCR) assay

The total RNA was extracted using TRIzol reagent (Invitrogen, Carlsbad, CA, USA). First-strand cDNAs were synthesized from 2 μg of the total RNA using a High-Capacity cDNA Reverse Transcription kit according to the manufacturer’s instructions (Invitrogen, Carlsbad, CA, USA). Quantitative real-time PCR was performed with Power SYBR Green PCR Master Mix (Life Technology, Foster City, CA, USA). Relative gene expression was determined by 2^–ΔΔCT^ method normalized to GAPDH^32^. The primers and reaction conditions are listed in Supplementary Table 2. All the samples were run in triplicate.

### Bisulfite genomic sequencing (BGS) and conversion-specific and methylation-specific polymerase chain reaction (BS-MSP) assay

Genomic DNA was extracted by a simplified proteinase K digestion method^33^. Bisulfite modification of genomic DNA was carried out as described previously using Epitect Fast Bisulfite Conversion Kits (Qiagen, Germany)^34^. The distribution of methylated CpG sites within CpG islands was first determined by BGS assay. The bisulfite-treated DNA was amplified using a set of BGS primers (Region 1: for ZNF667-AS1: from −1000 to −618 bp, for ZNF667: from 146 to 528 bp; Region 2: for ZNF667-AS1: from −200 to 71 bp, for ZNF667: from −543 to −273 bp; Region 3: for ZNF667-AS1: from 277 to 547 bp, for ZNF667: from −1019 to −749 bp), then cloned into pGEM-T vectors (Promega, Madison, WI, USA), with eight to ten clones randomly picked and sequenced by automatic fluorescence sequencing. According to the distribution of methylated CpG sites by BGS assay, the methylation status of three regions (Region 1: for ZNF667-AS1: from −925 to −744 bp, for ZNF667: from 272 to 453 bp; Region 2: for ZNF667-AS1: from −158 to −4 bp, for ZNF667: from −469 to −315 bp; Region 3: for ZNF667-AS1: from 326 to 479 bp, for ZNF667: from −951 to −798 bp) was then determined by BS-MSP method using methylation- or unmethylation-specific primer set. The BS-MSP products were analyzed on 2% agarose gel with ethidium bromide staining. The primers and reaction conditions for BGS and BS-MSP assays are listed in Supplementary Table 2.

### Luciferase reporter constructs and dual-luciferase reporter assay

To explore the effect of transcription factors and CpG sites methylation status on ZNF667-AS1 or ZNF667 transcriptional activity, promoter reporter plasmids of ZNF667-AS1 and ZNF667 were constructed. The amplified fragments were inserted into the pGL3-basic vector (Promega, Madison, WI, USA), and the recombinant plasmids were sequenced and confirmed. The pGL3-A1, pGL3-Z1, and pGL3-Z2 constructs were, respectively, in vitro methylated as described previously^35^. Eca109 cells (1 × 10^5^/per well) were inoculated in 24-well culture dishes 24 h before transfection. Two hundred nanograms of unmethylated or methylated ZNF667-AS1 or ZNF667 constructs, pGL3-control vector (positive control), or pGL3-basic vector (negative control) were then co-transfected with 10 ng of pRL-TK vector (Promega, Madison, WI, USA) using lipofectamine 2000 (Invitrogen, Carlsbad, CA, USA). After 48 h, luciferase activity was determined by Dual-Luciferase Reporter Assay System (Promega, Madison, WI, USA). The luciferase activity was expressed by the ratio of Firefly luciferase to Renilla luciferase activity.

### Chromatin immunoprecipitation (ChIP) assay

ChIP assay was performed using EZ-Magna ChIP A/G (17-10086, Upstate, Millipore, MA, USA) kit according to the manufacturer’s instructions. Antibodies against Sp1, E2F1, TET1, H3K27me3, UTX, or JMJD3 (4 μg per ChIP, Upstate, Millipore, MA, USA) were used for immunoprecipitation. Quantitative analysis of ChIP-derived DNA was performed by real-time qPCR analysis (primers in Supplementary Table 2). The assays were performed in triplicate.

### Cell transfection

For overexpression of ZNF667-AS1 or ZNF667, the cDNA encoding ZNF667-AS1 or ZNF667 was PCR-amplified and subcloned into pcDNA3.1 vector (Invitrogen, Carlsbad, CA, USA), named pcDNA3.1-ZNF667-AS1 or pcDNA3.1-ZNF667, respectively. The Eca109 and TE13 cells were, respectively, transfected with pcDNA3.1-ZNF667-AS1 or pcDNA3.1-ZNF667 expression plasmid or the empty vector (pcDNA3.1-NC) as control at a final concentration of 2 µg/µL using FuGENE HD Transfection Reagent (Promega, Madison, WI, USA). After transfection, the resistant cells were isolated using 800 μg/mL of G418 (Life Technologies, Carlsbad, CA, USA). For inhibition of ZNF667-AS1 or ZNF667, Kyse170 cells were, respectively, transfected with ZNF667-AS1- or ZNF667-specific shRNA plasmid using FuGENE HD Transfection Reagent, and a scrambled shRNA was used as a negative control. For overexpression of E2F1, TET1, UTX, and JMJD3, the cDNAs encoding them were PCR-amplified and subcloned into pcDNA3.1 vector (Invitrogen, Carlsbad, CA, USA).

### Cell viability assay

The viability of pcDNA3.1-ZNF667-AS1 or pcDNA3.1-ZNF667 transfected Eca109 and TE13 cells or shRNA transfected Kyse170 cells was determined by cell-counting kit-8 (CCK-8) assay following the manufacturer’s guidelines. Ten microliters of CCK-8 (Dojindo, Japan) were added to the 100 μl of cultured cells and after incubated for 2 h in a humidified incubator containing 5% CO_2_ at 37 °C, the absorbance value of each well was measured at 450 nm wavelengths.

### Soft agar colony-formation assay

For clone-formation assay, the transfected cells were regularly cultured for 1 week at 37 °C and 5% CO_2_. Surviving colonies were stained with crystal violet in 4% paraformaldehyde solution, with visible colonies (≥50 cells) counted under a microscope.

### Wound-healing assay

For wound-healing assay, a wound was made by a straight scratch with a 200-μL pipette tip in the cultured cells, and then captured the images at the same position of each well 0, 12, and 24 h after the wound was created under a microscope. The relative distance of cell migration to the scratched area was measured, and the cell-healing percentage was calculated.

### Cell invasion assay

The cell invasion assay was conducted using 24-well transwell chambers (Corning, Kennebunk, ME, USA) following the manufacturer's instructions. Briefly, the transwell chambers were coated with 30 μg of Matrigel (BD Biosciences, San Jose, CA, USA) and incubated at 37 °C for 1 h. The transfected cells in serum-free RPMI 1640 were loaded into the upper chambers. Then, RPMI-1640 medium supplemented with 10% FBS was loaded into the lower chamber. After 24 h incubation at 37 °C and 5% CO_2_, the number of cells that invaded through the filters were stained and counted in five microscopic fields (at ×100 magnification) per filter.

### Subcellular fractionation

To determine the cellular localization of ZNF667-AS1, Nuclear/Cytosol Fractionation Kit (BioVision, Milpitas, CA, USA) was used to collect the cytosolic and nuclear fractions of Kyse170 and TE1 cells (1 × 10^6^) following the manufacturer’s guidelines. The GAPDH gene was used as a cytoplasmic localization control, and U6 gene was used as a nucleus localization control.

### Western blot analysis

The total cell lysates were prepared in ice-cold RIPA lysis buffer followed by ultrasonication. BCA protein assay was used to determine the protein content, and 20 μg of protein lysates were separated by sodium dodecyl sulfate-polyacrylamide gel electrophoresis (SDS-PAGE) and transferred onto nitrocellulose filter membranes. The blots were first incubated with primary antibodies specific for ZNF667 (1:1000 dilution, rabbit anti-human polyclonal antibody, GeneTex, Alton Pkwy Irvine, CA, USA) or E-cadherin (1:1000 dilution, mouse anti-human monoclonal antibody, Abcam, UK), and then incubated with horseradish peroxidase-conjugated secondary antibody. The GAPDH (1:500 dilution, mouse anti-human monoclonal antibody, Abcam, UK) was used as a loading control.

### RNA immunoprecipitation (RIP) assay

RIP assay was performed using Magna RIP™ RNA-Binding Protein Immunoprecipitation Kit (Millipore, Billerica, MA, USA) following the manufacturer’s instructions. Antibodies against TET1, UTX, or JMJD3 (Upstate, Millipore, MA, USA) were used for immunoprecipitation. The IgG antibody was used as a negative control. The purified RNA was subjected to qRT-PCR analysis.

### RNA pull-down assay

LncRNA ZNF667-AS1 was in vitro transcribed using RiboMAX™ Large-Scale RNA Production Systems (Promega, Madison, WI, USA), biotin-labeled and purified with Pierce™ Magnetic RNA-Protein Pull-Down Kit (Thermo Scientific, Rockford, lL, USA) following the manufacturer’s instructions. Two milligrams of whole-cell lysates from cells were incubated with 50 pmol of purified biotinylated transcripts for 1 h at 4 °C; RNA-binding protein complexes were isolated with streptavidin magnetic beads; the proteins were separated by SDS-PAGE and detected by western blot analysis.

### hMeDIP-qPCR analysis

A simplified proteinase K digestion method was used to extract genomic DNA from cells^33^. The hMeDIP assay was performed as previously described^36^. Briefly, genomic DNA was denatured and then immunoprecipitated with anti-5hmC antibody or IgG control antibody and protein G magnetic Dynabeads (Invitrogen, Carlsbad, CA, USA). The beads were then treated with proteinase K, and the pulled-down DNA was analyzed by qPCR. The primers are listed in Supplementary Table 2.

### Statistical analysis

All data shown represent the results obtained from triplicated independent experiments with standard errors of the mean (mean ± SD). The real-time RT-PCR results were expressed as mean ± SD. Statistical expression differences between groups were determined using Student’s *t* test. The status of gene methylation between different groups was analyzed by Pearson’s Chi-square test. Kaplan–Meier method and the Log-rank or the Breslow tests were used to estimate overall survival. Cox’s multivariate test was used to adjust for potentially confounding variables and to evaluate the independent predictor of patients’ prognosis. Statistical analyses were performed using SPSS19.0 software package (SPSS Company, Chicago, Illinois, USA). All statistical tests were two sided; and *P* < 0.05 was considered statistically significant.

## Supplementary information

Supplementary figure 1

Supplementary figure 2

Supplementary figure 3

Supplementary table 1

Supplementary table 2

Supplementary table 3

Supplementary table 4

Supplementary table 5

Supplementary Figure legends
