# Supplementary figures and images for: Aberrant hypermethylation-mediated downregulation of antisense lncRNA ZNF667-AS1 and its sense gene ZNF667 correlate with progression and prognosis of esophageal squamous cell carcinoma
Source: Cell Death Dis. 2019 Dec 5;10(12):930. doi: 10.1038/s41419-019-2171-3 (PMC6895126; doi:10.1038/s41419-019-2171-3)

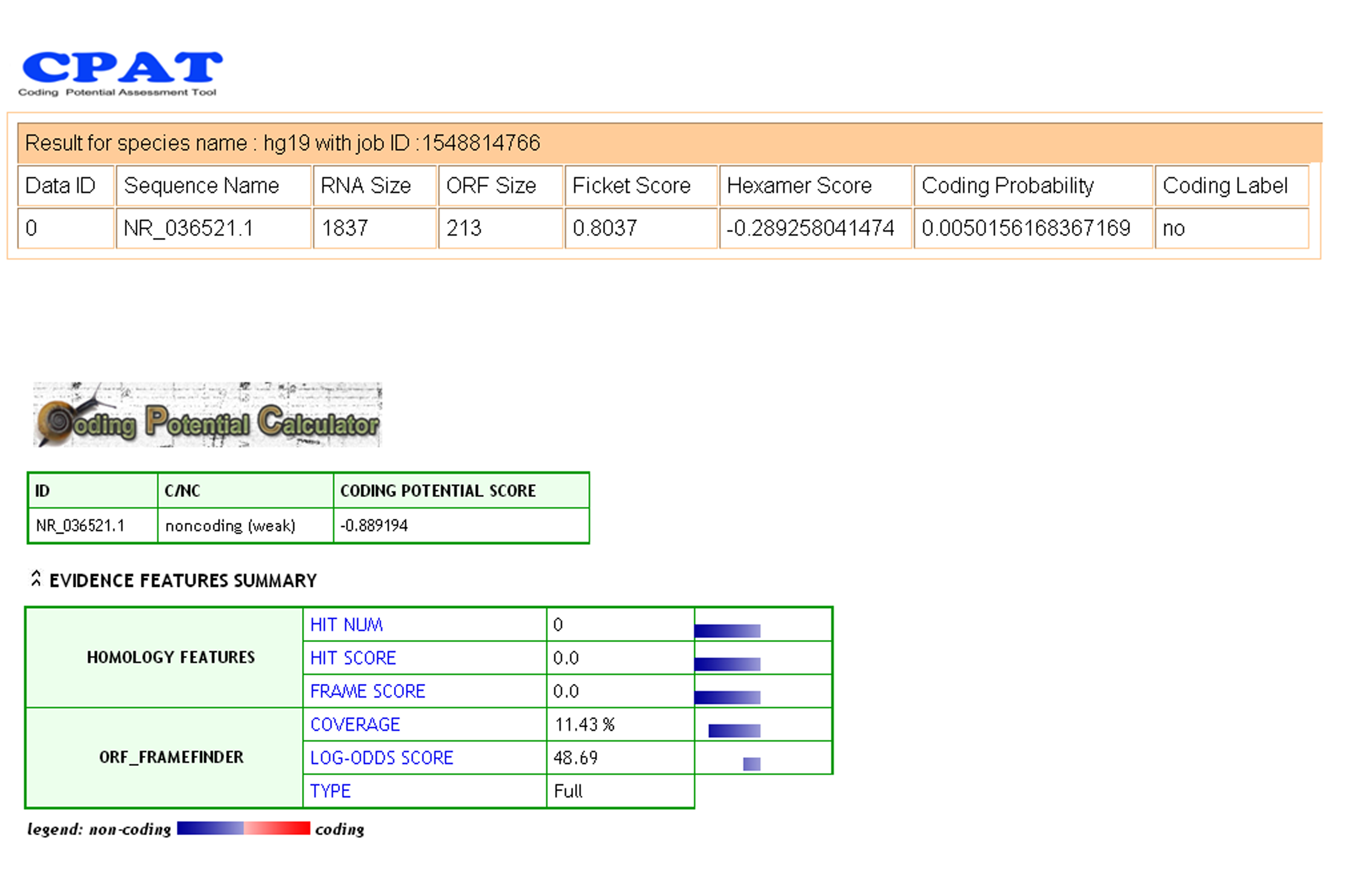

Supplement: Supplementary file 1 — Supplementary figure 1 [file 41419_2019_2171_MOESM1_ESM.tif]

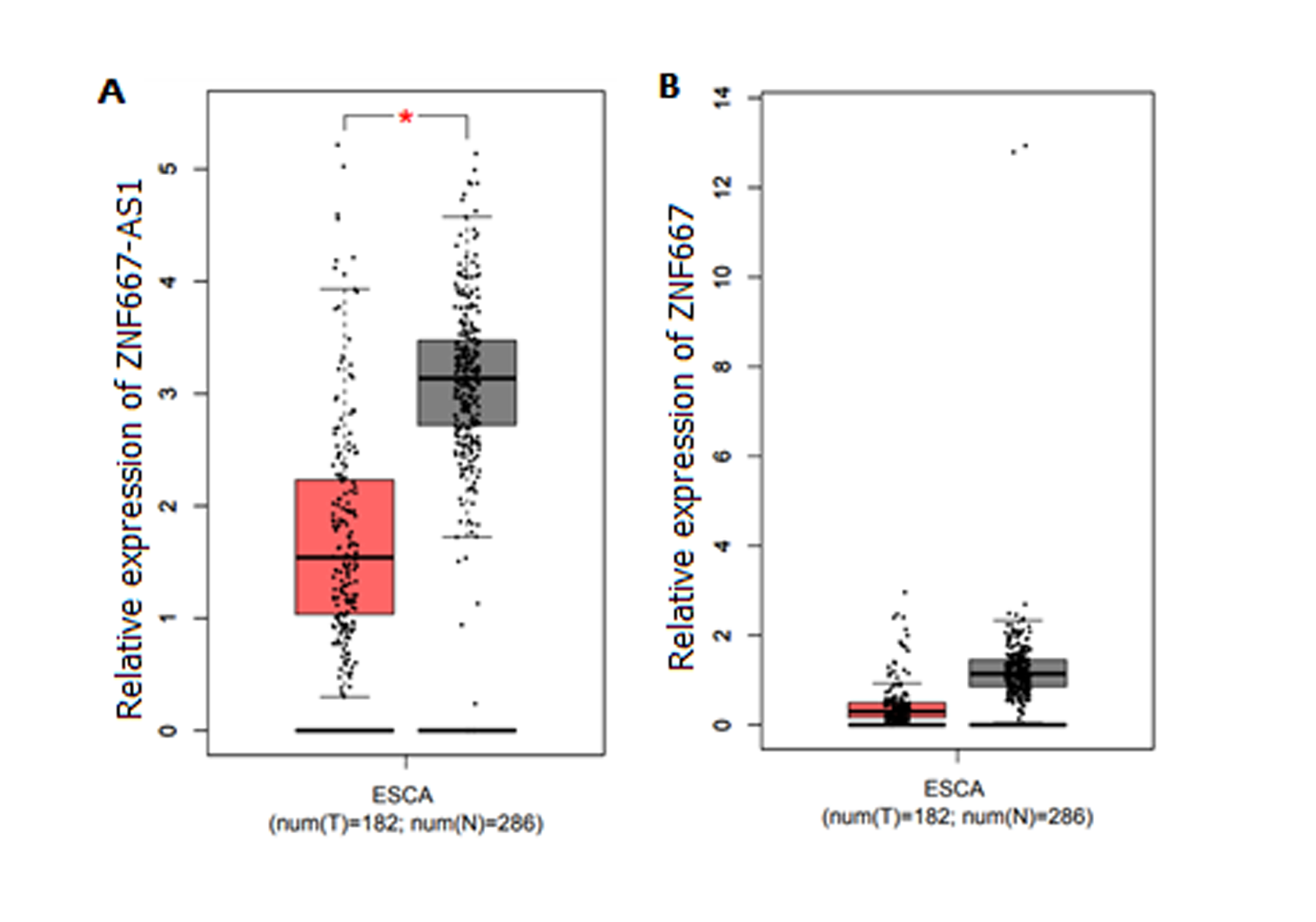

Supplement: Supplementary file 2 — Supplementary figure 2 [file 41419_2019_2171_MOESM2_ESM.tif]

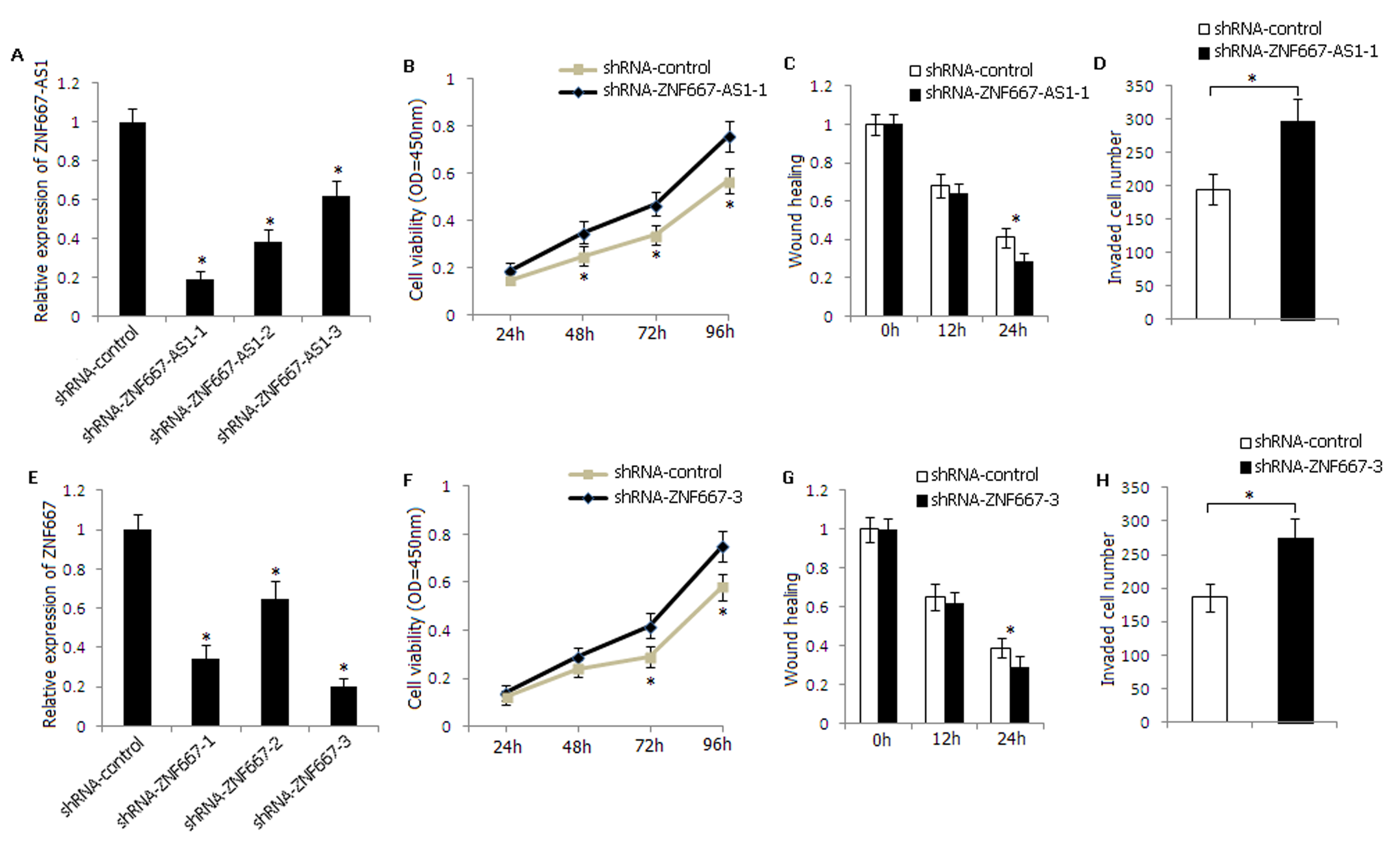

Supplement: Supplementary file 3 — Supplementary figure 3 [file 41419_2019_2171_MOESM3_ESM.tif]
