# Supplementary material for: Aberrant hypermethylation-mediated downregulation of antisense lncRNA ZNF667-AS1 and its sense gene ZNF667 correlate with progression and prognosis of esophageal squamous cell carcinoma
Source: Cell Death Dis. 2019 Dec 5;10(12):930. doi: 10.1038/s41419-019-2171-3 (PMC6895126; doi:10.1038/s41419-019-2171-3)
Supplement: Supplementary file 4 — Supplementary table 1 [file 41419_2019_2171_MOESM4_ESM.docx]

Table 1 Clinicopathologic characteristics of esophageal squamous cell carcinoma cases

| Groups | N (%) |
| --- | --- |
| Age |  |
| ＜60 | 62(45.9) |
| ≥60 | 73(54.1) |
| Gender |  |
| Male | 99(73.3) |
| Female | 36(26.7) |
| TNM stage |  |
| Ⅰ | 3(2.2) |
| Ⅱ | 75(55.6) |
| Ⅲ | 53(39.2) |
| Ⅳ | 4(3.0) |
| Pathological differentiation of tumor |  |
| Well | 27(20.0) |
| Moderate | 36(26.7) |
| Poor | 72(53.3) |
| Depth of invasion |  |
| T1/2 | 55(40.7) |
| T3/4 | 80(59.3) |
| LN metastasis |  |
| negative (N0) | 36(26.7) |
| positive (N1/2/3) | 99(73.3) |
| Distant metastasis or recurrence |  |
| negative | 78(57.8) |
| positive | 57(42.2) |
| Family history of UGIC |  |
| negative | 80(59.3) |
| positive | 55(40.7) |
| Vital statistics |  |
| Alive | 32(23.7) |
| Dead ESCC | 86(63.7) |
| Dead unrelated | 5(3.7) |
| Information unavailable | 12(8.9) |
