# Supplementary material for: Aberrant hypermethylation-mediated downregulation of antisense lncRNA ZNF667-AS1 and its sense gene ZNF667 correlate with progression and prognosis of esophageal squamous cell carcinoma
Source: Cell Death Dis. 2019 Dec 5;10(12):930. doi: 10.1038/s41419-019-2171-3 (PMC6895126; doi:10.1038/s41419-019-2171-3)
Supplement: Supplementary file 5 — Supplementary table 2 [file 41419_2019_2171_MOESM5_ESM.docx]

Table 2 Primer sequences and reaction conditions of ZNF667-AS1, ZNF667, and related genes used in this study

| PCR types | Genes | Primers | Annealing temperature (℃) | Product size (bp) |
| --- | --- | --- | --- | --- |
| qRT-PCR | ZNF667-AS1 | F:5’-CATCACTACCATCCATCACTA-3’ |  |  |
|  |  | R:5’-CCAGGCAGAGAAGGATAA-3’ | 59 | 186 |
|  | ZNF667 | F:5’-TGTGACAAGTTCTTCAGGCG-3’ |  |  |
|  |  | R:5’-GGATGAATGCCGATTGCAGAC-3’ | 60 | 140 |
|  | E-cadherin | F:5’-CGAGAGCTACACGTTCACGG-3 |  |  |
|  |  | R:5’-GGCCTTTTGACTGTAATCACACC-3' | 59 | 162 |
|  | GAPDH | F:5’-AGGTGAAGGTCGGAGTCAACG-3’ |  |  |
|  |  | R:5’-AGGGGTCATTGATGGCAACA-3’ |  | 104 |
| BGS | Region 1 | F:5’-TAGAAATATAAATTTTTATAGTTAG-3’ |  |  |
|  |  | R:5’-AAAATATAAATATATCTAAAAAC-3’ | 57 | 383 |
|  | Region 2 | F:5’-AGTTTTTTAATTAAATTTAATTT-3’ |  |  |
|  |  | R:5’-CAACCCAAACCTTCTTCACCTCA-3’ | 58 | 271 |
|  | Region 3 | F:5’-GGTTGGTAGTGAGATTGATTG-3’ |  |  |
|  |  | R:5’-TCCTCACACATATTCAAAATAAC-3’ | 56 | 271 |
| BS-MSP | Region 1 |  |  |  |
|  | First-step | F:5’-TAGAAATATAAATTTTTATAGTTAG-3’ |  |  |
|  |  | R:5’-AAAATATAAATATATCTAAAAAC-3’ | 57 | 383 |
|  | Second-step |  |  |  |
|  | Methylation | F:5’-TGGGTTACGTAGCGATATATATTCG-3’ |  |  |
|  |  | R:5’-TAAACTCACGCCTACGTAAATTCTCG-3’ | 57 | 182 |
|  | Unmethylation | F:5’-TGGGTTATGTAGTGATATATATTTG-3’ |  |  |
|  |  | R:5’-TAAACTCACACCTACATAAATTCTCA-3’ | 59 | 182 |
|  | Region 2 |  |  |  |
|  | First-step | F:5’-AGTTTTTTAATTAAATTTAATTT-3’ |  |  |
|  |  | R:5’-CAACCCAAACCTTCTTCACCTCA-3’ | 58 | 271 |
|  | Second-step |  |  |  |
|  | Methylation | F:5’-TCGTTTTTATAATTATACGTTTTCG-3’ |  |  |
|  |  | F:5’-AATCGCAAAATAACTACGTAATACG-3’ | 55 | 155 |
|  | Unmethylation | F:5’-TTGTTTTTATAATTATATGTTTTTG-3’ |  |  |
|  |  | F:5’-AATCACAAAATAACTACATAATACA-3’ | 51 | 155 |
|  | Region 3 |  |  |  |
|  | First-step | F:5’-GGTTGGTAGTGAGATTGATTG-3’ |  |  |
|  |  | R:5’-TCCTCACACATATTCAAAATAAC-3’ | 56 | 271 |
|  | Second-step |  |  |  |
|  | Methylation | F:5’-TTTATTTCGGCGGGGAGAAGGGACG-3’ |  |  |
|  |  | R:5’-CCCGCACGCCCAAATAACCGAAACG-3’ | 57 | 154 |
|  | Unmethylation | F:5’-TTTATTTTGGTGGGGAGAAGGGATG-3’ |  |  |
|  |  | R:5’-CCCACACACCCAAATAACCAAAACA-3’ | 59 | 154 |
| pGL3 plasmids | pGL3-A1 | F: 5'-TATAGATCTTGTGGACACTGAGGCCCTTC-3' |  |  |
|  |  | R:5'-GATAAGCTTCCCAAGCCTTCTTCACCTCA-3' | 58 | 310 |
|  | pGL3-A2 | F: 5'-TATAGATCTCACACCCACACTCTCGCCAG-3' |  |  |
|  |  | R:5'-GATAAGCTTCCCAAGCCTTCTTCACCTCA-3' | 58 | 717 |
|  | pGL3-A3 | F:5'-CAGAGATCTTTCACAGTCAGCGTCACACA-3' |  |  |
|  |  | R:5'-GATAAGCTTCCCAAGCCTTCTTCACCTCA-3' | 56 | 1072 |
|  | pGL3-A4 | F:5'-CAGAGATCTTTCACAGTCAGCGTCACACA-3' |  |  |
|  |  | R:5'-TATAAGCTTGTCTGAGGGCGTGGGCGGCG-3' | 57 | 374 |
|  | pGL3-A5 | F:5'-TATAGATCTGCTGCGCTGGTTCTGCTCTG-3' |  |  |
|  |  | R:5'-GATAAGCTTCTACACAAACGCGCGATCAA-3' | 56 | 459 |
|  | pGL3-Z1 | F:5'-CATAGATCTGGAAGGGCCTCAGTGTCCAC-3' |  |  |
|  |  | R:5'-TATAAGCTTACTCAGCGCCTGCGCACTCC-3' | 58 | 362 |
|  | pGL3-Z2 | F:5'-GATAGATCTCGGACAGAGCAGAACCAGCG-3' |  |  |
|  |  | R:5'-TATAAGCTTACTCAGCGCCTGCGCACTCC-3' | 56 | 656 |
|  | pGL3-Z3 | F:5'-GCTAGATCTAGGTTCGTCCTCACACATG-3' |  |  |
|  |  | R:5'-TATAAGCTTACTCAGCGCCTGCGCACTCC-3' | 56 | 1119 |
|  | pGL3-Z4 | F:5'-GCTAGATCTAGGTTCGTCCTCACACATG-3' |  |  |
|  |  | R:5'-CATAAGCTTGGGTCGGAGATGAACTGACC-3' | 57 | 482 |
|  | pGL3-Z5 | F:5'-TATAGATCTTCCCTGCTCGGCTGTCTCGG-3' |  |  |
|  |  | R:5'-TAGAAGCTTCACCCACTCCCGGCTTCCCA-3' | 57 | 440 |
| ChIP-qPCR | ZNF667-AS1 site 2 | F:5'-TTCCTCGAGCTCTTTAACCA-3' |  |  |
|  |  | R:5'-GCTGGCTGGAGGCAATAAGC-3' | 58 | 101 |
|  | ZNF667-AS1 site 5 | F:5'-ACATTCACGAGGTTTGGAGC-3' |  |  |
|  |  | R:5'-ATTCTGGAGGTCGCAGGATG-3' | 57 | 94 |
|  | ZNF667 | F:5'-GGATGACTGCGTAGTGCG-3' |  |  |
|  |  | R:5'-CACAACCACACGTTCCCGT-3' | 56 | 141 |
|  | E-cadherin | F:5’- ACTCCAGGCTAGAGGGTCAC-3’ |  |  |
|  |  | R:5’- CGTACCGCTGATTGGCTGA-3’ | 57 | 130 |
| hMeDIP-qPCR | ZNF667 | F:5'-GGATGACTGCGTAGTGCG-3' |  |  |
|  |  | R:5'-CACAACCACACGTTCCCGT-3' | 56 | 141 |
|  | E-cadherin | F:5’- ACTCCAGGCTAGAGGGTCAC-3’ |  |  |
|  |  | R:5’- CGTACCGCTGATTGGCTGA-3’ | 57 | 130 |
| F: Forward primer; R: Reverse primer | | | | |
