# Supplementary material for: Aberrant hypermethylation-mediated downregulation of antisense lncRNA ZNF667-AS1 and its sense gene ZNF667 correlate with progression and prognosis of esophageal squamous cell carcinoma
Source: Cell Death Dis. 2019 Dec 5;10(12):930. doi: 10.1038/s41419-019-2171-3 (PMC6895126; doi:10.1038/s41419-019-2171-3)
Supplement: Supplementary file 6 — Supplementary table 3 [file 41419_2019_2171_MOESM6_ESM.docx]

Table 3 Methylation status of three regions in ESCC tumor tissues and corresponding normal tissues

| Group | N | Methylation frequency | | | | | |
| --- | --- | --- | --- | --- | --- | --- | --- |
|  |  | Region 1 | | Region 2 | | Region 3 | |
|  |  | n (%) | P | n (%) | P | n (%) | P |
| Normal tissues | 135 | 46(34.1) |  | 15(11.1) |  | 28(20.7) |  |
| Tumor tissues | 135 | 104(77.0) | <0.001 | 82(60.7) | <0.001 | 107(79.3) | <0.001 |
