# Supplementary material for: Aberrant hypermethylation-mediated downregulation of antisense lncRNA ZNF667-AS1 and its sense gene ZNF667 correlate with progression and prognosis of esophageal squamous cell carcinoma
Source: Cell Death Dis. 2019 Dec 5;10(12):930. doi: 10.1038/s41419-019-2171-3 (PMC6895126; doi:10.1038/s41419-019-2171-3)
Supplement: Supplementary file 7 — Supplementary table 4 [file 41419_2019_2171_MOESM7_ESM.docx]

Table 4 Methylation status of three regions in ESCC tissues

| Group | N | Methylation frequency | | | | | |
| --- | --- | --- | --- | --- | --- | --- | --- |
|  |  | Region 1 | | Region 2 | | Region 3 | |
|  |  | n (%) | P | n (%) | P | n (%) | P |
| Age |  |  |  |  |  |  |  |
| ＜60 | 62 | 51(82.3) |  | 42(67.7) |  | 51(82.3) |  |
| ≥60 | 73 | 53(72.6) | 0.184 | 40(54.8) | 0.125 | 56(76.7) | 0.428 |
| Gender |  |  |  |  |  |  |  |
| Male | 99 | 78(78.8) |  | 63(63.6) |  | 79(79.8) |  |
| Female | 36 | 26(72.2) | 0.423 | 19(52.8) | 0.253 | 28(77.8) | 0.798 |
| TNM stage |  |  |  |  |  |  |  |
| Ⅰ+ Ⅱ | 78 | 53(67.9) |  | 36(46.1) |  | 56(71.8) |  |
| Ⅲ + Ⅳ | 57 | 51(89.5) | 0.003 | 46(80.7) | <0.001 | 51(89.5) | 0.012 |
| Pathological differentiation |  |  |  |  |  |  |  |
| Well/moderate | 63 | 41(65.1) |  | 28(44.4) |  | 45(71.4) |  |
| Poor | 72 | 63(87.5) | 0.002 | 54(75.0) | <0.001 | 62(86.1) | 0.036 |
| Depth of invasion |  |  |  |  |  |  |  |
| T1/2 | 55 | 40(72.7) |  | 30(54.5) |  | 41(74.5) |  |
| T3/4 | 80 | 64(80.0) | 0.324 | 52(65.0) | 0.222 | 66(82.5) | 0.263 |
| LN metastasis |  |  |  |  |  |  |  |
| negative (N0) | 36 | 26(72.2) |  | 17(47.2) |  | 28(77.8) |  |
| positive (N1/2/3) | 99 | 78(78.8) | 0.423 | 65(65.7) | 0.052 | 79(79.8) | 0.798 |
| Distant metastasis or recurrence |  |  |  |  |  |  |  |
| negative | 78 | 58(74.4) |  | 44(56.4) |  | 61(78.2) |  |
| positive | 57 | 46(80.7) | 0.387 | 38(66.7) | 0.228 | 46(80.7) | 0.724 |
| Family history of UGIC |  |  |  |  |  |  |  |
| negative | 80 | 61(76.3) |  | 47(58.8) |  | 62(77.5) |  |
| positive | 55 | 43(78.2) | 0.793 | 35(63.6) | 0.568 | 45(81.8) | 0.543 |
