# Supplementary material for: Aberrant hypermethylation-mediated downregulation of antisense lncRNA ZNF667-AS1 and its sense gene ZNF667 correlate with progression and prognosis of esophageal squamous cell carcinoma
Source: Cell Death Dis. 2019 Dec 5;10(12):930. doi: 10.1038/s41419-019-2171-3 (PMC6895126; doi:10.1038/s41419-019-2171-3)
Supplement: Supplementary file 8 — Supplementary table 5 [file 41419_2019_2171_MOESM8_ESM.docx]

Table 5 Multivariate analysis of survival in ESCC cases (Cox’s test)

| Variable | B | SE | P | Odds ratio (95%CI) |
| --- | --- | --- | --- | --- |
| TNM stage | 1.587 | 0.283 | <0.001 | 4.890 (2.806-8.522) |
| Pathological differentiation | 0.751 | 0.261 | 0.004 | 2.119(1.270-3.534) |
| LN metastasis | 1.266 | 0.398 | 0.001 | 3.547(1.626-7.734) |
| Family history of UGIC | 0.741 | 0.237 | 0.002 | 2.098(1.318-3.341) |
| ZNF667-AS1 expression | 0.335 | 0.261 | 0.199 | 1.398(0.838-2.331) |
| ZNF667 mRNA expression | -0.320 | 0.261 | 0.219 | 0.726(0.436-1.210) |
| Region 2 methylation | 0.655 | 0.262 | 0.012 | 1.926(1.153-3.217) |
