# Supplementary material for: Aberrant hypermethylation-mediated downregulation of antisense lncRNA ZNF667-AS1 and its sense gene ZNF667 correlate with progression and prognosis of esophageal squamous cell carcinoma
Source: Cell Death Dis. 2019 Dec 5;10(12):930. doi: 10.1038/s41419-019-2171-3 (PMC6895126; doi:10.1038/s41419-019-2171-3)
Supplement: Supplementary file 9 — Supplementary Figure legends [file 41419_2019_2171_MOESM9_ESM.docx]

Figure S1 Coding potential of ZNF667-AS1 predicted by Coding Potential Assessment Tool and Coding Potential Calculator.

Figure S2 The relative expression level of ZNF667-AS1 and ZNF667 in esophageal carcinoma (ESCA) in GEPIA datasets.

Figure S3 Influence of down regulation of ZNF667-AS1 and ZNF667 on Kyse170 cells proliferation, migration, and invasion.
